# Macroeconomic and health care aspects of the coronavirus epidemic: EU, US and global perspectives

**DOI:** 10.1007/s10368-020-00465-3

**Published:** 2020-05-23

**Authors:** Paul J. J. Welfens

**Affiliations:** 1 European Economy and International Economic Relations (EIIW), University of Wuppertal, Rainer-Gruenter-Str. 21, D-42119 Wuppertal, Germany; 2 AICGS/Johns Hopkins University, Washington DC, USA

**Keywords:** Coronavirus, Health system, Macroeconomics, EU, US, China, I11, I18, F01, H51

## Abstract

The novel coronavirus (COVID-19) epidemic represents a major challenge for the world economy. While a detailed longer-term diffusion path of the new virus cannot be anticipated for individual countries, one may anticipate international supply shocks and declining GDP growth in many OECD countries and China in 2020; and one should expect falling asset prices in Asia, the United States and the European Union plus the United Kingdom – except for the price of risk-free government bonds. In the course of 2020/21 the US, the EU and the UK, as well as other countries, will face both an increasing number of infected patients as well as a higher case fatality ratio. Health care expenditures in the US could increase more than in the Eurozone and the EU in the medium term, a development that undermines the international competitiveness of the United States. The analysis suggests that per capita income is a positive function of the effective trade openness and of the new Global Health Security Index indicator from the NTI/Johns Hopkins University. A rising health care-GDP ratio in the US is equivalent to a rising US export tariff. As regards the coronavirus challenge, the ratio of acute care beds to the elderly in OECD countries shows considerable variation. Due to international tourism contraction alone, output growth in the Eurozone, the US and China can be expected to fall by about 1.6% in 2020. The COVID-19 challenge for the US Trump Administration is a serious one, since the lack of experts in the Administration will become more apparent in such a systemic stress situation – and this might well affect the November 2020 US presidential election which, in turn, would itself have considerable impacts on the UK and the EU27 as well as EU-UK trade negotiations. Integrating the health care sector into macroeconomics, which should include growth analysis, is an important task. The role of health quality - and health insurance coverage - for endogenous time horizons and economic welfare, respectively, is emphasized.

## Introduction

The novel coronavirus (for short COVID-19, or COrona VIrus Disease 2019) epidemic which started at the end of 2019 in the Wuhan area of China has, within three months, affected about 90,000 people worldwide of which approximately 3000 have died. The number of countries reporting infections has increased rapidly and the high case fatality rate has caused many individuals, firms and governments to react in various ways in order to try to limit the spread of the virus. In the EU and the US, this has included imposing quarantine on people who have recently returned from abroad – for example, from China – or who have participated in certain social events (such as the carnival celebrations in western Germany in late February, where in one location alone, the city of Heinsberg which is close to Düsseldorf, many people seem to have contracted the virus) in which individuals who have tested positive for COVID-19 had also participated and who have been on the radar of health authorities. Many firms in Germany and France have encouraged employees to practice home office and thus have tried to minimize infection risks within the company; other countries, including the US, have followed in March 2020. Such adjustment measures in firms, while pragmatic, will go along with reduced labor productivity and innovation, but at least a lot of important work can still be done remotely. The authorities imposed a so-called “lockdown” in Italy in mid-March and other EU countries have followed with similar measures in order to slow down the spreading of the coronavirus.

In car factories – for example Volkswagen – workers have raised the question of why production should continue meaning that workers are exposed to a spreading infection while white collar workers are sitting at home with the family following the government-imposed restrictions in many western OECD countries in mid-March, namely to avoid situations with many people congregating in the same area. Problems with lack of intermediate products supplied from abroad could thus slow down industrial production as much as workers’ fear of the propagation of the coronavirus and the spread infection at the factory floor, respectively. To the extent that regulations have been imposed to close hotels and restaurants both domestic and international tourism are extremely restricted in the first and second quarter of 2020. Thus the question arises which macroeconomic effects one has to anticipate and how the effectively massive epidemic test of the health system and the hospital sector, respectively, will cause in the many countries affected.

The first part of this contribution is an introduction and an overview of key issues. The basic idea of the whole contribution is to argue that a broader and deeper analytical link between macroeconomic approaches and health system analysis seems to be adequate; indeed required if one is to largely understand the unique medium- and long-term effects of the coronavirus shock – or of similar future shocks. Moreover, some new key indicators for the capacity of the hospital sector in the coronavirus pandemic are presented for OECD countries. The usefulness of the recently available Global Security Health Index is emphasized and analytical findings of the augmented Mundell tradables/non-tradables model are presented along with a simple modified Solow growth model which includes the health insurance coverage ratio as a critical variable in the context of an approach with an epidemic shock. A crucial aspect of this contribution is to emphasize established analytical positive links between health and productivity and output growth, respectively – generally emphasized by, amongst others, Mushkin (1962), Hsiao (2000) and for the case of China by the empirical findings of Li and Huang (2009). The subsequent analysis is based on knowledge available at March 24, but the theoretical approaches highlighted and developed here should be useful beyond changes in the CORVID-19 statistics.

### Some key figures on the pandemic

COVID-19 is spreading worldwide and on March 11, 2020, the World Health Organization (WHO) defined the international epidemic to be of a global nature: COVID-19 was by that date officially regarded as a pandemic. With 104 countries affected, about 420,000 people infected and nearly 19,000 fatalities (for an overview see the map of the WHO as of March 25, 2020^1^ and tables in Appendix 1 Tables 7 and 8). As of mid-March, 146 countries were affected, 154,000 people had been infected and 5700 fatalities had been recorded. In absolute terms, the number of infections was very high in China with 81,048 cases, followed by Italy (21,157), Iran (12,729), Republic of Korea (8162), Spain (5753), France (4469), Germany (3795), US (1678), Switzerland (1359) and the UK (1144). Given the nature of the pandemic, the picture quickly changes: For March 23, 2020, the WHO reported 81,601 cases in China which is roughly a stagnation, but Italy already had recorded 59,138, Spain 28,572, Germany 24,774 and France 15,821 cases. The age distribution of coronavirus-related deaths for China shows that the elderly have, not surprisingly, a higher mortality than the average. One economic implication is that, on the one hand, the effective labor force should be negatively affected by COVID-19. On the other hand, there is an unclear effect on retired persons and the overall death rate as the shut-down of firms improves the overall air quality and thus could reduce the number of people falling ill from non-coronavirus diseases (Fenz and Kharas 2020). With hospitals facing full capacity utilization in acute care capacities, the survival rate of other inpatients and outpatients with serious illnesses (non COVID-19) could fall. As regards the WHO figures, one may note that the oft-cited figures published by the Johns Hopkins University differ due to the broader statistical coverage by the latter.

The only country in the top seven countries where the apparent e-function type related expansion was different – starting from the day when 100 infections had been reported – was the Republic of Korea where broad testing and strict quarantine measures had been applied. Another country with a rather low number of infections reported was Taiwan (with less than 50 confirmed cases by March 9, 2020) where government relied on a ready emergency plan built upon the previous experience of the SARS epidemic and shut down all air travelling with China early on.

**Map 1:**
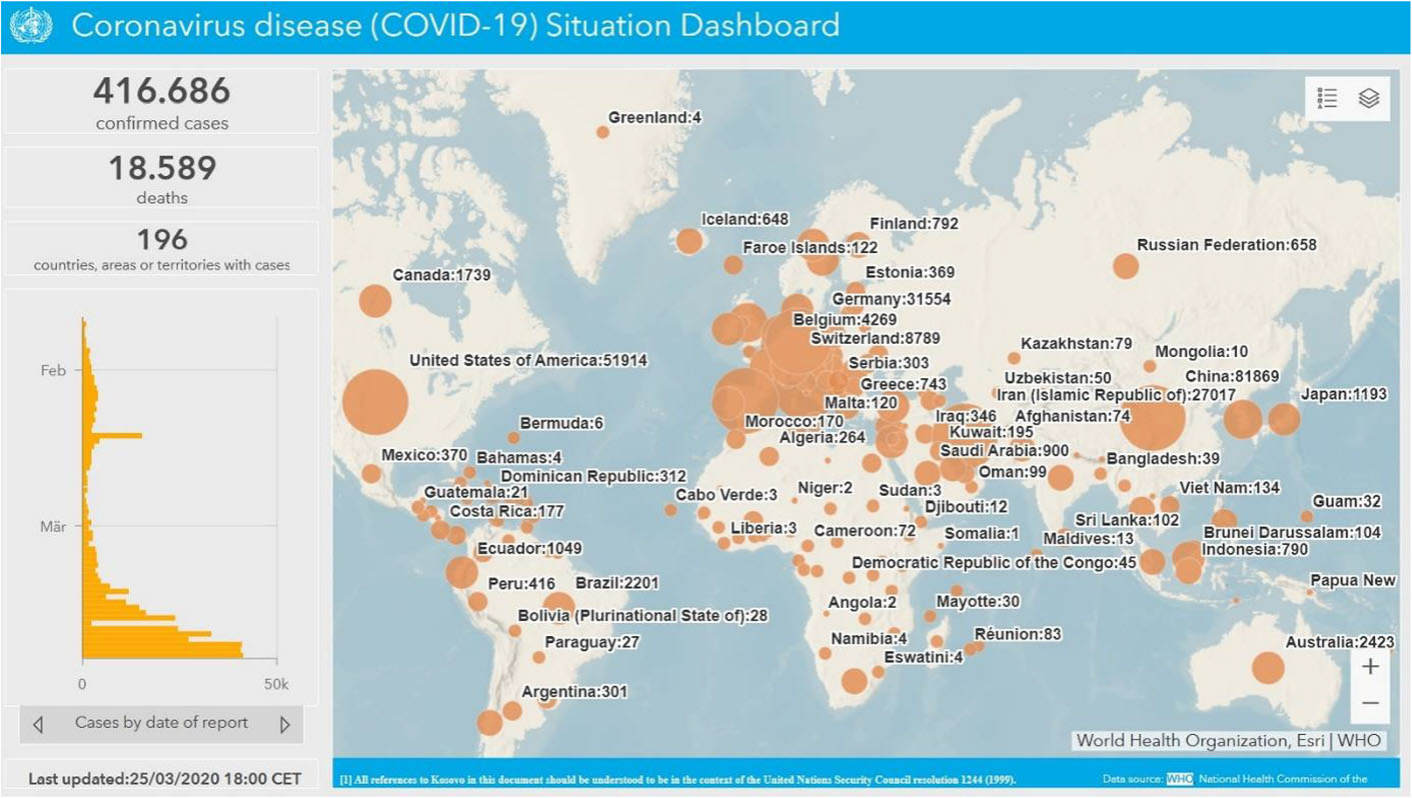
World Health Organization map of the 2019 Coronavirus Spreading Worldwide (as of March 25, 2020) Source: Map extracted from https://experience.arcgis.com/experience/685d0ace521648f8a5beeeee1b9125cd (last accessed 26.03.2020)

The pandemic could bring a minor recession for OECD countries and China or several quarters of much reduced output in industry and the services sector. International tourism will be very modest in 2020 and might not recover before the second half of 2021. In a normal recession, tourism – representing a large economic sector in many countries – is usually not much affected. A lack of vacation time in turn and the stress from the threat of the pandemic might also depress considerable strata of society for some time so that productivity could decline strongly for many months. As PSA, Renault, Fiat-Chrysler, Volkswagen and other EU car producers have announced on March 16 that production in several plants will be shut down for several months in Europe a deep recession cannot be excluded, as there could be a unique overlap of declining manufacturing output and services sector contraction, including tourism. There is also the risk that the normal chain of payments could be interrupted as firms or households face serious liquidity constraints – and firms or banks’ solvency constraints - if the economic crisis deepens. A deepening economic crisis will make it more difficult to bolster the resources allocated to the health sector. The hospital sector is obviously crucial to the response to the coronavirus epidemic and here the indicators are critical for many countries, including many EU countries, namely when acute care beds relative to the elderly population are considered (see subsequent sections). This contribution brings together macroeconomic and health system aspects in a somewhat unusual way, but this perspective could be useful for the analysis of the coronavirus epidemic (Map 1).

An epidemic typically starts with a small diffusion of the number of people infected, after a few months or quarters there will be a peak as counter measures by government as well as individuals - and physicians in the health system - have been implemented. After the peak has been reached, the number of infected will gradually fall. From this perspective, the macroeconomic effects in a first stage of an epidemic should be rather modest, followed by a peak of negative output shocks – finally followed by a potentially enhanced economic upswing as postponed investment and consumption in the private sector could increase. Both mortality and morbidity statistics published should affect aggregate demand on the one hand, but also aggregate supply on the other hand as there will be less people working in factories and offices in an epidemic period. Of particular relevance for the control of the spatial spreading of COVID-19 are measures which effectively impose a quarantine on those who are infected; and for many other people, the authorities could impose restrictions on mobility at the regional, the national and the global level. Many people, including tourists and business people, will be eager to reduce their level of international travel, particularly to regions/countries with high infection problems. From this perspective, China – where COVID-19 started in late 2019 – has a specific problem, but one should also notice that China has had growing numbers of visitors, including business people, and tourists in the two decades after 1990 (see Appendix Fig. 9).

A pandemic like COVID-19 is a crucial shock to most national health systems in countries with a high number of infections and is also a shock to the world economy. Given the fact that health care expenditures relative to gross domestic product in industrialized countries are between 8 and 18% in 2019, it seems crucial to link health care expenditure analysis to the more traditional macroeconomic analysis. Indeed, there is a lack of such analysis as has already been noted by Gerdtham and Jonsson (2000) in their classical health care analysis contribution “International Comparison of Health Expenditure: Theory, Data and Econometric Analysis” in the Handbook of Health Economics. The coronavirus shock to the world economy is a case where indeed an overlap of macroeconomic analysis and health care analysis indeed makes a lot of sense. To the extent that the COVID-19 shock reduces real income for several quarters in many countries, one should also see a medium term decline of health care expenditures (after the short-term transitory peak determined by the epidemic) since the demand for health care rises over-proportionately with real income.

The statistics on China’s regional incidence of COVID-19 infections show that two provinces are primarily affected which suggests that the drastic quarantine measures imposed by the national and regional authorities seem to have been effective. As regards the EU, Italy is a hotspot with more than 7000 confirmed cases at the end of the first week of March. The US Center for Disease Control was reporting eleven fatalities in the US on March 7, 2020, while the WHO) were documenting no COVID-19 related deaths in the United States on the same day (the WHO figures were subsequently updated in the following days).

On March 24, the WHO had already registered a total of 42,164 coronavirus infections in the United States, including 10,591 new infections and 471 fatalities. New York seemed to be a regional hot spot and on March 24 the Trump Administration declared that visitors to New York should undergo 14 days in self-isolation. Given the fact that New York had 64 million US visitors plus 14 million foreign visitors plus an unknow number of New York people who visited friends or business colleagues and other institutions in the US, and indeed outside the US, the New York cluster indeed stands for a crucial challenge. Assuming that 500,000 US visitors had been in New York as visitors in the two weeks prior to March 24, about half a million US citizens should go into quarantine and the roughly 100,000 foreign visitors who had visited New York in the same period should also followed the advice for a self-imposed quarantine. However, these people apparently have not been contacted although airlines could have been mobilized to track foreign visitors and to alert them about the need for self-quarantine. This is an example of how poorly organized the world economy is in facing the challenge of a pandemic and how negligent authorities in the US and outside the US have dealt with the fact that New York is a coronavirus hot spot. While the very nature of a pandemic calls for global economic cooperation, it seems that such cooperation is at a premium in 2020; to what extent political populism contributes to this problem could be an important aspect in future research.

### Early assessments of the coronavirus Epidemic’s economic effects

As regards the economic impact of COVID-19 in various countries, one may point out that it is not only direct and indirect channels into the real economy which will be relevant, but changes in income expectations which will (this includes digital news channels – e.g. the diffusion of COVID-19 related information in the internet) affect the behavior of investors and consumers as well as policymakers. Psychological effects on the demand side could play a strong role in the current epidemic and negative effects on the aggregate demand side could overlap with supply-side disturbances from international problems in the delivery of intermediate inputs. Liquidity problems on the side of firms could also contribute to an economic slowdown, moreover liquidity problems of major banks might occur in many countries so that output decline in OECD countries as well as in China and other countries could be considerable. In early March 2020, McKibbin and Fernando (2020) have presented a macro model with various scenarios for the world economy which show a large range of negative possible real GDP outcomes in the context of the COVID-19 challenge, including a major international recession.

The Interim Economic Assessment of the OECD (2020) from March 2, 2020 has argued that global output growth could decline to a low rate of 2.4%, down from the 2.9% of 2019 – but in 2021, the output growth would rise to 3.3% in the world economy (see following Table 1). The interim assessment of the OECD (2020) showed in March only slight negative effects on output in 2020 and 2021, respectively; the peak of the infection was assumed to be in the first quarter 2020. In a special simulation case with a peak only in the second half of 2020, a stronger output decline is shown, namely −1.75% relative to the baseline; North America records −1.5% Table 1.

At the end of the first quarter of 2020, it is still too early to fully assess the output decline from the coronavirus shock, but the order of magnitude for western OECD countries could come close to the output decline related to the Transatlantic Banking Crisis. The type of shock represented by the coronavirus pandemic is normally not considered in standard macroeconomic simulations – hence the combined effect of a shutdown of the tourism sector and the hospitality sector plus the automotive sector plus other services (say, a barber shop closed for several weeks as a consequence of epidemic-related government regulations – with no relative price adjustment able to bring about a new market equilibrium in the short term) first have to appear on the analytical radar of simulation expert groups. The duration of the demand effect is rather uncertain in the early stage of the pandemic, but even once the duration becomes clear after a few months there could be serious balance sheet effects on companies with limited working capital and short-term debt maturities – hence, even a temporary closure of firms can have serious effects in some economic sectors. It is thus not clear that after a few months of the temporary shutdown of many firms, one could more or less reset the whole economy and continue output expansion on the old level of the growth path. Since the pandemic shock affects nearly all countries of the world economy there is also an unusual global synchronization of the shock and the big fiscal packages considered in the US, the EU, the UK and in other countries clearly imply a medium-term rise of debt-GDP ratios in OECD countries and many newly industrialized countries. Developing countries without much room to maneuver will need strong support by the IMF, the World Bank and regional multilateral banks. The coronavirus pandemic thus is a challenge that requires a multilateral response. Multilateralism, however, is not the favored approach of the Trump Administration with its emphasis on bilateralism. By implication, there will be a lack of US leadership in this international economic crisis that in turn also could raise doubts about standard open economy simulation results.

In a typical DSGE macro model the strong output decline after the initial epidemic shock is followed by a later output increase where part of the initial output loss is compensated by higher output growth in the following quarters. As long as the coronavirus challenge can be overcome in a medical sense by autumn 2020, postponed consumption and investment should be useful to contribute to a new economic upswing in 2021 and beyond; an adequate mix of monetary policy and fiscal policy can contribute to overcoming the incipient recession. To the extent that China, the US, the EU27 plus the UK and Japan adopt similar expansionary policy, there will be international spillover effects on the one hand which reinforces the upswing. However, quantitative easing (QE) which normally brings about a nominal and real devaluation, and a decline of the interest rate, will not bring much output expansion through the real devaluation impulse if several big countries adopt similar QE policies.

As regards the UK, it is, however, clear that the expansionary policy mix adopted in spring 2020 means that there is not much room to maneuver for a strong fiscal and monetary impulse to cushion the BREXIT shock. The UK in turn faces sharper problems in the health system post-BREXIT – as of February 1, 2020; the UK’s access to special medical programs of the EU are no longer available and the number of foreign workers in the UK National Health Service (NHS) has declined in the period 2016–2019. This is a problem in a period in which demand stress on intensive care in the NHS hospital system will increase. The number of hospital beds per inhabitant in the UK is rather low by an international comparison amongst OECD countries, at the same time, one may point out that Italy, Spain and France had recorded high number of COVID-19 infections in March 2020.

**Table 1:** Interim Economic Outlook (EO), Year-on-Year Percentage Change in Real GDP Growth Forecasts for Selected Countries/Economies (OECD 2020)

		2020		2021	
Countries	2019	2020 Interim EO	Difference from November EO	2021 Interim EO	Difference from November EO
World	2.9	2.4	−0.5	3.3	0.3
G20	3.1	2.7	−0.5	3.5	0.2
Australia	1.7	1.8	−0.5	2.6	0.3
Canada	1.6	1.3	−0.3	1.9	0.2
Euro Area	1.2	0.8	−0.3	1.2	0.0
Germany	0.6	0.3	−0.1	0.9	0.0
France	1.3	0.9	−0.3	1.4	0.2
Italy	0.2	0.0	−0.4	0.5	0.0
Japan	0.7	0.2	−0.4	0.7	0.0
Korea	2.0	2.0	−0.3	2.3	0.0
Mexico	−0.1	0.7	−0.5	1.4	−0.2
Turkey	0.9	2.7	−0.3	3.3	0.1
United Kingdom	1.4	0.8	−0.2	0.8	−0.4
United States	2.3	1.9	−0.1	2.1	1.1
Argentina	−2.7	−2.0	−0.3	0.7	0.0
Brazil	1.1	1.7	0.0	1.8	0.0
China	6.1	4.9	−0.8	6.4	0.9
India	4.9	5.1	−1.1	5.6	−0.8
Indonesia	5.0	4.8	−0.2	5.1	0.0
Russia	1.0	1.2	−0.4	1.3	−0.1
Saudi Arabia	0.0	1.4	0.0	1.9	0.5
South Africa	0.3	0.6	−0.6	1.0	−0.3

Source: Own representation based on table in OECD (2020), p. 2. Note: G20 aggregate does not include the EU, and projections are based on data available to February 28, 2020

**Table 2:**
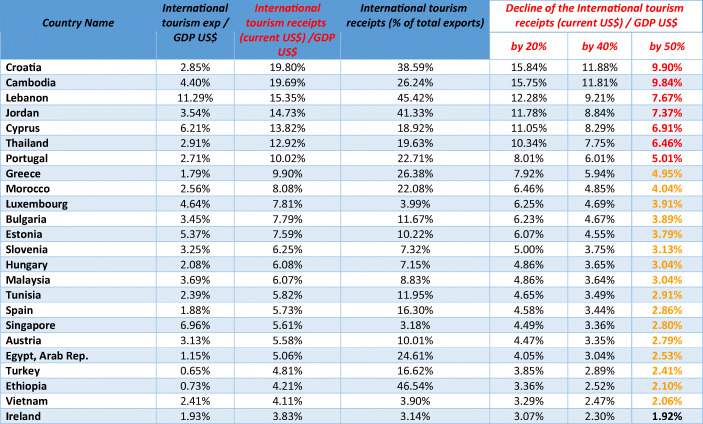
Selected Countries Strongly Affected by a Decline in International Tourism Receipts (based on appendix and the underlying calculations; direct real GDP effects in the last column)

Source: Own representation of data from the World Development Indicators and own calculations

**Table 3:**
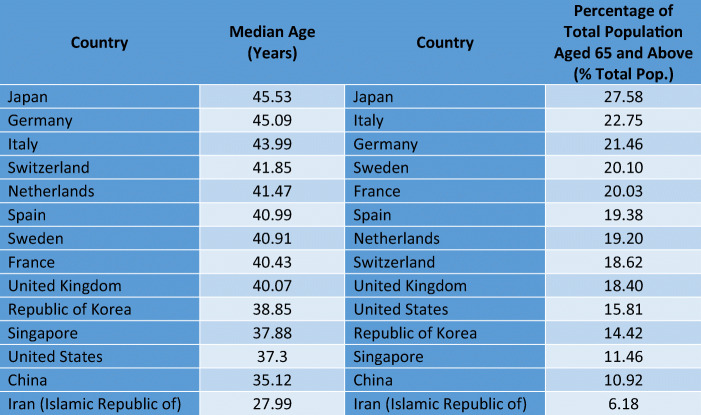
Median Age and Percentage of Total Population Aged 65 and over in Selected Countries, 2018

Source: Own representation of data; data for the median age from UN https://data.un.org data for 2012; data for percentage of the total population over 65 from World Bank, World Development Indicators, data for 2018; ranked highest to lowest

**Table 4:** Regression for Real Per Capita GDP (PPP) Figures: Cross Country Analysis for 2018 (174 countries; list of countries and data source: see Appendix 7 Table 12)

	(1) ln_gdppc	(2) ln_gdppc	(3) gdppc	(4) gdppc
ghs	0.0395*** (0.00395)	0.0402*** (0.00398)	623.3*** (82.62)	652.3*** (82.10)
ifdi_trueo~n	−1.279 (1.191)		−55,470.3 (34,015.1)	
trade_true~n	0.735*** (0.113)	0.721*** (0.110)	16,624.0*** (3711.1)	15,966.6*** (3820.4)
fdi _trueopen		−1.394 (1.055)		−34,391.2 (31,443.0)
_cons	7.759*** (0.199)	7.714*** (0.205)	−4808.6 (3844.5)	−6025.0 (4004.5)
N	174	174	174	174
R-sq	0.475	0.476	0.465	0.455
adj. R-sq	0.465	0.467	0.456	0.446
rmse	0.848	0.847	16,205.2	16,357.4

Standard errors in parentheses

*p < 0.05, **p < 0.01, ***p < 0.001

Source: Own representation. For the full list of countries and the data sources, please see Appendix 7 Table 12

**Table 5:** Hospital Beds per 1000 Population and Acute Care Hospital Beds per 1000 Population for OECD Countries

Country	Hospital Beds per 1000	Country	Acute Care Beds per 1000
Japan	13.05	Japan	7.79
Korea	12.27	Korea	7.14
Germany	8	Germany	6.02
Austria	7.37	Lithuania	5.47
Hungary	7.02	Austria	5.45
Czech Republic	6.63	Belgium	5
Poland	6.62	Slovak Republic	4.91
Lithuania	6.56	Poland	4.85
France	5.98	Hungary	4.27
Slovak Republic	5.82	Slovenia	4.2
Belgium	5.66	Czech Republic	4.11
Latvia	5.57	Luxembourg	3.77
Estonia	4.69	Greece	3.6
Luxembourg	4.66	Switzerland	3.56
Switzerland	4.53	Estonia	3.45
Slovenia	4.5	Latvia	3.3
Greece	4.21	Portugal	3.25
Australia	3.84	Norway	3.2
Norway	3.6	France	3.09
Portugal	3.39	Netherlands	2.92
Netherlands	3.32	Finland	2.8
Finland	3.28	Turkey	2.78
Italy	3.18	Ireland	2.77
Iceland	3.06	New Zealand	2.69
Israel	3.02	Italy	2.62
Spain	2.97	Denmark	2.54
Ireland	2.96	Iceland	2.51
Turkey	2.81	United States	2.44
United States	2.77	Spain	2.43
New Zealand	2.71	Israel	2.2
Denmark	2.61	United Kingdom	2.11
United Kingdom	2.54	Sweden	2.04
Canada	2.52	Chile	1.99
Sweden	2.22	Canada	1.96
Chile	2.11	Mexico	1.38
Mexico	1.38		

Source: Own representation of data from OECDStat, Health Care Resources, data for 2017 or latest available year; no data available for acute bed capacities for Australia

By mid-March 2020, the European Central Bank, the Bank of England and the US Federal Reserve System had all reduced interest rates or adopted – as with the ECB which was already operating at a zero interest rate level – more favorable conditions for banks to get central bank loans. It is not clear that monetary policy is suitable to counter-balance the negative effects of the COVID-19 pandemic whose economic disturbance is largely a supply-side disturbance (shocks to international production networks) and liquidity problems of many firms as well as some aggregate demand weakening. Fiscal expansion packages had also been adopted in the US, the UK and some leading EU countries by mid-March 2020. The European Commission (2020) emphasized that a consistent expansionary policy mix could help to stabilize the EU in a situation in which supply-shocks and demand shocks – including a negative shock from China in the first quarter of 2020 - as well as liquidity problems of firms were overlapping. One may add that the EU and the EU countries, respectively, had been passive in seeking restrictions on air travel to and from China in February and March 2020 which probably was not helpful in containing the spreading of the epidemic to Europe; by contrast the US imposed flight travelling restrictions with China in February.

While China and other countries in Asia are facing the epidemic as a crucial challenge for the health system and the political as well as the economic system Western Europe and the United States try to anticipate the spreading of the virus and to develop an adequate response in health policy, economic policy and in the field of international cooperation. As regards China, the province of Hubei (with the epidemic center of Wuhan) was very strongly affected by COVID-19 and the Chinese authorities have largely closed down production in the region, but schools and universities across the whole region have also undertaken quarantine measures. Authorities in China have closed down production in several regions which implies that firms in Europe, the US and Asia face a shortage of intermediate inputs from China; negative demand effects in China and in other countries could also be observed. Sales of cars fell in February 2020 by about 80% compared to the previous month before which clearly indicates a case of a strong negative sectoral demand shock.

One key intermediate export of China are computer chips which are imported by companies in the US, Europe, Asia, Latin America, Australia and Africa. The first sector facing a reduction in production after Chinese export slowdown will be computer and mobile phone producers as well as producers of modern screens. In a second round of supply-chain transmissions, digital service providers would obviously have to slow down planned expansion of such services and this in turn would reduce productivity growth in OECD countries and Newly Industrialized Countries. If Chinese firms can restore production capacity rather quickly, the negative supply-side effects for other countries should be rather modest, but if there is a second wave of COVID-19 in China, the global supply side shock of COVID-19 could be rather big. Taking into account the digital productivity slow-down in the world economy this shock would come on top of sectoral declines in tourism and logistics.

As regards the response from International Organizations and multinational firms to the COVID-19 outbreak in China, they typically recalled international personnel located in Beijing (and other Chinese centers) home in early and mid-February. European, American as well as Japanese firms in many cases followed the example of international organizations; those coming back to EU28 countries or the US were expected to implement a 14 days self-isolation in home quarantine.

While many observers of the COVID-19 epidemic – and politicians in the US, the EU and China/Japan/Republic of Korea - raise questions related to national health system challenges, there is not much awareness that the novel coronavirus with its potential as a worldwide epidemic (a pandemic) concerns a global public evil; and fighting the virus in these and other countries stands for a global public good. It is obvious that fighting a global public evil requires cooperation among the leading economies and in the relevant international organizations (e.g. the World Health Organization, the International Monetary Fund, the World Bank, UNHCR – in the case of refugees). In this perspective, the fact that the world’s global technological leader, the United States, is governed by the Trump Administration which refutes multilateralism might become a serious problem. Fighting a pandemic is a global public good and if there are considerable political free rider problems, or simply political inconsistencies and inefficiencies in major OECD countries, the fight against the global epidemic will be not really successful. This in turn implies that many more lives could be lost than in the case of efficient and effective global cooperation.

The fact that the world economy is facing the challenge of COVID-19 as a global problem in early 2020 could mean that the world economy is facing an instability problem, namely to the extent that the output decline in 2020/21 will seriously affect more than one half of the world economy: The disease emerged in China, standing for about 17.5% of world real income and thus a bit more than the US and the EU28 with each representing 16.5% in 2018 (PPP figures according to the World Bank). If national and international epidemic shocks translate into a serious economic slowdown in China in 2020, it will automatically have major negative international spillovers to the EU and the US and from these two actors there will be a strong negative repercussion effect on China. In short, a COVID-19 pandemic in the new world economy of triadic interdependency EU-US-China, requires enhanced international cooperation and multilateralism; but the Trump Administration is emphasizing bilateralism which means that the efficiency of OECD countries+China fiscal/monetary policy cannot be efficient. This in turn makes fighting the pandemic more difficult since an economic downturn in the global North would undermine economic stability and prosperity in the global South which implies that the problem of insufficient resources in the health sector of developing countries would be reinforced. The very nature of a pandemic is, however, such that the South of the world economy could be directly affected by the COVID-19 shock, and relatively poor countries – with rather weak public health systems – could face massive problems in dealing with the health care challenges as well as with the economic effects from this shock.

If the G20 countries would, in the end, face a simultaneous COVID-19 problem – outside China the peak of the pandemic may still be expected to occur only in summer 2020 (and possibly a second wave in autumn 2020 or thereafter) – there could be a global recession, as the G20 stands for 81% of global GDP. If the diffusion of COVID-19 can be stopped rather quickly, there is no major reason to worry with respect to the output and job development in the world economy, but if the pandemic should go on until 2021 or even beyond, there could emerge a very serious global stability problem. Given the pandemic and the likely size of the economic shock in sectors such as tourism and logistics – plus economic multiplier effects - policymakers should potentially be rather concerned in North America, Europa, Asia and other regions of the world economy.

Moreover, as regards the EU27/Eurozone and the US it will be interesting to take a closer look at the one sector which is directly exposed to the pandemic, namely the health care sector. The size and characteristic of that sector in the EU and the US clearly justifies the argument that this is a systemically relevant sector. To the extent that the health care sector and the economy – with health insurance linked to firms in the US – are characterized by inefficiencies, the COVID-19 challenge will reveal those inefficiencies to a considerable extent.

If there is a person with a suspected infection, a test is typically necessary and if the result of that test is positive, the respective person must stay in quarantine at home or go to hospital. If patients exhibit a serious reaction to COVID-19, they will typically be taken care of as in-patients in hospitals where strict quarantine conditions and protective measures for the people working there are necessary. The US Congress has appropriated an extra $8 billion in early March to increase the health care budget in the context of COVID-19 cases. Italy has introduced an additional package to the value of €7 billion in an extra budget on March 5, 2020, in order to fight the challenge posed by the virus. Additional fiscal packages were adopted in mid-March in the US, Germany, France and Italy.

The very high mortality rate in Italy in early 2020 suggests that the number of infected people in Italy has been underestimated. This raises questions about the quality of the Italian health system and health policy in Italy, respectively. On March 9, 2020, the Italian government imposed a lockdown on the whole country, while Austria a week later had signaled that it wants to restrict free travel between Italy and Austria (which are both Schengen area countries).

By March 17, most EU countries had imposed new border control measures which were supposed to control and restrict travelling of people across borders in the EU as a means to reduce the corona virus infection rate. However, an economically serious side effect was that long queues of trucks at many borders soon occurred which inevitably was bound to undermine just-in-time production in the automotive industry in most EU countries. It is quite unclear why the new border controls were not installed in such a way that the supply-side chains in the EU single market were not seriously disrupted: The avoidable massive slow-down of truck-based logistics in the EU amounts to a grave delivery risk for automotive firms whose temporary closing is thereby caused (plus the impulse of reduced demand). This part of the economic recession risk facing the EU should and could have been avoided by coordinated efficient policies of EU countries. There is a high probability that this effect will bring a higher economic burden related to the COVID-19 crisis for the EU countries compared to the United States where such problems do not exist.

Employees of firms in many countries have cancelled planned meetings in Italy and tourism in Italy is also bound to suffer considerably in 2020. As regards German and French car producers, as well as producers of machinery and equipment in Germany and France, firms in both countries partly rely on intermediate inputs from Italy so that distortions of relevant production in Italy will also slow down industrial output in Germany and France. This situation will, of course, encourage firms to seek alternative intermediate product suppliers. As regards a comparison of the US and the EU, European firms are more dependent on international intermediate inputs than firms in the US (Welfens and Irawan 2014). A general problem for the US, the EU, China and all other countries with novel coronavirus problems is that vaccination against the COVID-19 will probably not be available at short notice.

International investors have responded to the COVID-19 shock: Stock market prices have declined steeply in mid-March and are likely to further fall in the medium term. The very fact that there is a pandemic means that almost all countries in the world economy will face similar problems: Supply-side distortions, demand shocks and liquidity problems faced by firms and millions of self-employed. This makes the corona virus economic crisis potentially worse than the 2008/09 Transatlantic Banking Crisis. However, due to many reforms in banking regulation and institutional innovations undertaken since that crisis, the western world is better prepared for an international shock than it was in late 2008. Moreover, there is some probability that once the infection wave can be stopped – after a few quarters (or possibly after two years) – there will be a quick economic recovery. The potential for supply disruptions is, however, considerable since global value-added chains have strongly evolved in certain sectors since the 1990s.

As regards the US, the Eurozone/UK and China, there is one specific distinction concerning the western world (e.g. US+Eurozone+UK + Switzerland) versus China, namely that safe haven effects can be expected in a period of an international epidemic – indeed in favor of the US and main Eurozone countries such as Germany, France, the Netherlands and Austria; plus the UK and Switzerland. These countries should benefit from lower nominal and real interest rates, but should also face a nominal and real appreciation of the currency.

The following section considers the SARS experience briefly and emphasizes that certain characteristics of the health systems of the US and EU countries have crucial macroeconomic effects that have thus far not been thoroughly considered in Economics. Section 3 considers a cross country regression with the Global Health Security Index as an explanatory variable for per capita GDP. Section 4 is devoted to theoretical macroeconomic aspects of the COVID-19 epidemic, while Section 5 considers some financial market aspects. Section 6 briefly considers political economy aspects in the western world, while Section 7 provides a growth modelling approach which looks at more long-term issues of an epidemic. The final section considers implications for policymakers. The following analysis puts the analytical focus on some new theoretical perspectives which should be useful for the understanding of the coronavirus related economic dynamics and potential policy responses.

## Economic disruptions: Tourism sector shock and other key epidemic aspects

The travel and tourism sector will be negatively affected by the COVID-19 pandemic; this sector stood for 10.4% of global GDP and 319 million jobs in 2018 (WTTC 2019). If the global tourist sector declines by 30% in 2020, global output growth would decline by 1.2 points compared to forecasts - and expectations - of 2019 and 96 million jobs would be lost as a direct effect. By March 6, 2020, the airline Lufthansa had decided to cancel 7000 flights scheduled for 2020 which is about 50% of all flights: With a strong focus on flights to China, Republic of Korea, Italy and Iran which all are countries with high number of infections. Air France and other EU carriers adopted similar restrictions.

The share of tourism in national output in selected countries is shown in the following table. Countries with a high share of tourism in national output should expect high output growth dampening effects. However, one should not overlook the aspect that French people, for example, who would normally go on vacation abroad will instead book a vacation within France – thus replacing part of the normally large incoming international tourist groups from many countries. Hence popular tourist destination countries have some opportunities to adjust for the declining international tourism. The internet creates many opportunities to substitute international visits of business people. Trade fair events can also partly be organized as a virtual event if necessary. However, it is useful to consider scenarios of a contraction of international tourism value-added by 20%, 40% and 50% (see following table). For Germany, France, Italy (and the UK), a 50% decline brings a GDP decline of about 1%; for Italy, this would imply a recession in 2020. The decline of expenditures in tourism broadly defined – including entertainment (restaurants etc.) – would raise the negative output effect furthermore. As regards tourism receipts relative to GDP in EU countries, Switzerland and Turkey, high figures were in Bulgaria (6.8%), Estonia (5.8%), Greece (8.7%), Spain (5.7%), Croatia (18.4%), Cyprus (13.9%), Luxembourg (7.0%), Malta (12.7%), Portugal (8.3%), Slovenia (5.9%), Switzerland (3.9%) and Turkey (50% according to Eurostat, see Appendix 3 Table 9); as regards the statistics on Turkey, one may assume that the figure is doubtful. It is clear that countries such as Greece, Cyprus, Malta and Portugal might face new problems as a consequence of a dramatic decline in tourism expenditures in the context of a coronavirus pandemic, the same applies to Turkey.

There are two countries which could have strong improvements in the current account balance from the net effect of COVID-19 on receipts and expenditures in tourism. In 2018, German expenditures stood at €80.9 billion, while receipts were €36.4 billion (balance -€44.5 billion), so that a relatively strong decline of international tourist expenditures – with additional substitution effects in favor of higher domestic tourism expenditures – should reinforce the current account balance of the Eurozone. A similar effect could be expected in the UK which had a net balance of -€17.3 billion in 2018 (for more details, see table in Appendix 3 Table 9). For the US, a 50% contraction of international tourism would bring about an output decline of 0.6% as a direct effect. As the subsequent table shows, there are many small countries which would face serious output contractions in the case of a 50% decline of international tourism: There is a group of countries who could have an output decline of over 10%, and for the Lebanon, which at the beginning of 2020 was already in an unstable fiscal and economic situation, the projected output decline would be −7.67%; for Jordan the output decline expected is 7.37%, followed by Cyprus, Thailand and Malta with −6.91%, −6.46% and − 6.43%, respectively. The output decline for Croatia would be 9.90%, for Portugal 5.01%, for Greece 4.95% and for Spain 2.86%; thus there is a risk that the Euro Crisis could return (for more countries, see following Table 2 and Appendix 4 Table 10).

It should be noted that domestic tourism was still possible in Western Europe in the first half of March 2020. However, from about March 15 on, the lockdowns imposed in many EU countries effectively eliminated the option of domestic tourism for several weeks. Hence the whole tourism sector is facing an almost 100% shutdown for at least a few months.

Historically, there were previous cases of international epidemics (pandemic is a worldwide epidemic), such as the Spanish influenza in 1918/1919, the Asian influenza in 1957 and the Hong Kong influenza in 1968 (Kilbourne 2006). In the severe Spanish influenza, between 30 and 60 million people succumbed to the disease worldwide. Bell and Lewis (2004, p. 159) argue that no firm conclusions have been achieved on the long run effects of international epidemics.

The authorities have focused on reducing the number of, or avoiding the holding of, public events with many people as well as the interaction of many people in any given place - in the Wuhan area, factories and workplaces were closed over several weeks. In many countries, quarantine was imposed on people who have returned from China and on people who have shown COVID-19 symptoms. The infection typically brings respiratory problems and the elderly in many of the countries affected indeed seem to face a rather high mortality rate. As COVID-19 affects the lungs of the infected, regions/countries with bad air quality and high shares of smokers should go along with a relatively high mortality rate; weak environmental policy thus could translate into particularly serious COVID-19 problems. This coronavirus is, however, not the first epidemic of the early twenty-first century. In 2003, SARS (or Severe Acute Respiratory Syndrome) – the outbreak of which was also traced back to China - was the first international epidemic of the century, followed by MERS (Middle East Respiratory Syndrome) which mainly affected some countries in the Middle East.

With the outbreak of COVID-19, the world economy is clearly facing transitorily lower economic growth in 2020 than had been projected in autumn 2019 (based, for example, on the IMF World Economic Outlook). The IMF has declared on March 4 (IMF 2020) that it will make an additional $50 billion in funding available to member countries fighting the coronavirus with particular funding reserved for rather poor countries.

In the EU and the Eurozone, respectively, Italy – actually Northern Italy - had been most affected by COVID-19 by the end of February 2020. It is not fully clear why Italy in particular is facing so many cases of infections and a relatively high mortality rate. Looking at health system quality indicators thus seems to be useful and the subsequently discussed indicator of the NTI/Johns Hopkins University study – the Global Health Security Index (https://www.ghsindex.org/wp-content/uploads/2019/10/2019-Global-Health-Security-Index.pdf) - is considered to be an adequate aggregate indicator with several useful sub-indicators: The aggregate index has a clear focus on epidemic risks and the quality of the respective national health system; the indicator shows a large variation across countries in the EU, the OECD and G20 countries, respectively. There are some links between this indicator and macroeconomic development, including the following:

- A high score in the Global Health Security Index could be interpreted as a quality signal by foreign investors for whom often high quality health provision for managers in the host country, as well as a good health system for the workers employed in the subsidiary abroad, are important aspects to consider in the context of international investment and locational choice for greenfield investment projects or international M&As.
- Not only does the quality of the respective health system matter but also the efficiency of the health system and hence cost aspects – indirectly visible in tax rates and social security contribution rates – of production. Countries with rather inefficient health systems have a specific problem in cost competitiveness; certainly in labor intensive industries. The United States has, somewhat surprisingly, some specific problems in this field that have gone almost unnoticed by most international macroeconomists for many years. In the context of the COVID-19 epidemic, which had already reached the US in late February 2020, the inefficiencies of the US health system could become visible again.

For the health systems of the respective countries and regions, respectively, the coronavirus epidemic is a particular challenge; as in any epidemic scenario, there are particular risks that physicians and nursing personnel could be infected, and hospitals as well as nursing and care institutions for the elderly are potential hot spots in terms of infection risk. Special clothing, masks and disinfection measures should typically protect the life of physicians and nursing personnel. At the same time, there are standard plans and approaches of protection and treatment aimed at controlling infections (quarantines of affected individuals of a few weeks being one of the standard measures) and the spreading of the virus. However, with masks and other protective devices in stock in only limited numbers, an international spreading of the virus can quickly lead to shortages; for example, at the beginning of March 2020, the French government seized all available stocks of medical masks nationally and forbade the exporting of masks in the fear of having an under-supply of masks if such medical goods would be exported in considerable number. In Germany, authorities imposed similar restrictions in the first week of March.

Epidemics can have grave negative consequences on local, national or international demand. In the case of the SARS epidemic, for example, tourism in Hong Kong reduced by 90% in two months in the first quarter of 2003. A study on the case of a serious epidemic in the US by the US Congressional Budget Office (CBO 2005) thus also assumes considerable negative demand effects from an epidemic which, of course, would affect many people and which would also have a certain fatality rate. In a similar EU-related simulation study on the macro effects of an epidemic, Jonung and Roeger (2006) adopt a similar analytical approach, but also assume a permanent negative shock to population growth (−0.75%).

As regards the preparedness of countries to deal with the present coronavirus epidemic, it is interesting to consider the results of the analysis of the NTI/Johns Hopkins University (2019) – for more GHS Index rankings, see Appendix 5 Table 11 - which shows various elements of preparedness of countries to deal with an epidemic. The leading country in the relevant Global Health Security Index, according to this study is the US, with a No. 1 ranking in the aggregated overall indicator, other OECD countries and some Newly Industrialized Countries are also in the group of leading countries. The UK and the Netherlands are ranked No. 2 and No. 3, respectively, in the aggregate Health Security Indicator, France is ranked No. 11, and Germany No. 14 in the aggregate indicator. Developing countries typically have low rankings in the overall indicator and the sub-indicators. Thailand stands out among the Newly Industrialized Countries with a favorable position in the aggregate indicator (No. 6) and in some sub-indicators. In the aggregated indicator, China is ranked No. 51, India is on No. 57; while the Russian Federation occupies 63rd position, Romania and Bulgaria are No. 60 and 61, respectively – even further behind is, surprisingly, Luxembourg (No. 67). Clearly, few economic experts are thus far aware of these critical rankings in the Global Health Security Index first published in late 2019; rankings which highlighted certain weak points in the European Union.

As of early March 2020, it is obvious that international tourism and passenger air transportation are negatively affected by the coronavirus epidemic. Given the fact that more than 50% of global trade is trade in intermediate products, there are also shocks to international supply chains. Moreover, trade fairs and sports events have been cancelled so that also hotels, restaurant and other related services have been negatively affected. As several countries/regions have closed schools and universities, the education system is also negatively affected. It is clear that the closing of production facilities in several regions of Asia will lead to distortions in terms of Asia-EU and Asia-US supply chains so that there is a supply shock to firms in the tradables sector in the EU and the US, respectively.

With firms in the services sector starting to lay off workers, and with a more pessimistic economic perception on the part of households and investors, aggregate demand is slowing down in spring 2020 on the one hand, on the other hand the demand in the non-tradables sector (services) in particular can be expected to fall. The main effect of the epidemic thus is:

- A negative supply shock in the tradables sector in the short and medium term
- A negative demand shock in the tradables sector and in the non-tradables sector (the negative demand shock in the non-tradables sector might dominate initially, partially due to a sharp contraction of demand in tourism and entertainment) in the short term; once a vaccination becomes available in OECD countries and G20 countries, respectively, demand growth should become positive again.
- A negative aggregate international demand shock in the medium term which could stem from both reduced consumption and investment postponement effects – with many countries generating parallel negative spillover effects in neighboring countries and with main trading partners, respectively; China is a top trading partner of both the US and many EU countries, thus the outbreak of COVID-19 in China has affected very many OECD countries.

The analytical perspective on the macroeconomics of the COVID-19 is straightforward and shown subsequently in this paper. Basically, as regards policy responses, one may want to consider three aspects:

- The supply-side response of government in the health system; for example, governments buying extra quantities of medical equipment and medicines which should drive up prices in the respective sectors.
- Monetary policy, which mainly concerns the US, the Eurozone, the UK and China. In a small open economy, an expansionary monetary policy under fixed exchange rate would be not effective; only fiscal policy would work – and it could work if government can finance some deficit spending with a clear focus on the non-tradable sector (e.g. construction activities for infrastructure).
- Fiscal policy, which mainly concerns the US, EU countries, the UK and China plus other Asian countries exposed to the virus shock; Thailand, for example, normally has a rather high number of Chinese tourists and business people visiting every year, but with the problem of COVID-19 in China, these visits will decline dramatically and Thailand could decide to adopt an expansionary fiscal policy. A similar logic could hold for other ASEAN countries as well. To the extent that certain countries in Asia are effectively fixing the exchange rate vis-à-vis the US dollar, a macro analysis in a fixed exchange rate system would be adequate.

In a significant policy step, the US Federal Reserve System reduced the interest rate in early March 2020 by 0.5 percentage points; followed by another interest rate reduction by 1 percentage point only about a week later. It is not clear that expansionary monetary policy is adequate to cope with a negative supply-side shock. The US interest rate reduction will stimulate aggregate demand in the US and, in this context, could also stimulate net exports of goods and services through a real depreciation of the currency. At the same time, the associated real appreciation of the Euro will dampen aggregate demand in the Eurozone; and a similar argument will hold with respect to China whose currency appreciation would dampen China’s GDP. The dampening of output in both China and the Eurozone will dampen US aggregate output through a dampening effect on US net exports.

In the Eurozone, the European Central Bank (ECB) does not have considerable room to maneuver and possibly welcomes the FED’s interest rate reduction (plus potential new quantitative easing measures in the US) since this will also reduce the interest rate in the Eurozone. Given the fact that in the US, the UK and the Eurozone interest rates are already very low, there is some risk that a reversal interest rate effect will occur (Brunnermeier and Koby 2018) which could dampen aggregate output as a reduction of the interest rate brings a reduction of banks’ profits from the deposit business which could compensate the valuation gains the banks experience with high interest legacy bonds in the banks’ balance sheets. The ability of banks to extend loans could critically depend on net-worth – once this constraint becomes binding – and hence lower central bank interest rates would bring about a decline of loans to firms and the real economy, respectively; traditional monetary policy is no longer expansionary. As regards the ECB, it still has some room to maneuver despite a zero central bank interest rate (and negative deposit rates for banks) as the ECB could step up quantitative easing – with the potential problem of having to go above the current upper limit of 1/3rd of outstanding government bonds – and it could also give more long term conditional loans to banks at favorable interest rates, namely under the condition that banks would extend more loans to firms. It is, however, not fully clear what the medium-term purpose of such a measure should be if this would raise the excess supply of the tradables sector in the Eurozone and the EU, respectively – there is some risk that this would depress the global level of tradables prices, a development which, in turn, could destabilize the world economy in the medium term.

Expansionary fiscal policy could also be considered in the US, the EU (the EU countries) and China. Given the interdependency of the US, the EU and China, fiscal policy coordination would be adequate, but it is unclear what institution could/should be the platform to achieve this type of coordination. The US-Sino trade conflict has at least been moderated somewhat through the US-China trade agreement in early February 2020 so that some bilateral coordination of fiscal policies of the US and China is not excluded. Some coordination between the US and the EU countries could take place via the OECD, but given the competence gap in the Trump Administration in the Treasury, this might be difficult to achieve: The Trump Administration could fill only about 3000 of the roughly 4000 political appointee roles which became vacant at the end of the previous Obama Administration, which means that the Trump Administration suffers from a lack of about 1000 experts in key fields (Welfens 2019) – and the Treasury is a key institution exposed here. The G20 as a coordination platform is rather excessive and overly complex, so that one might consider the OECD’s outreach program, which includes China and India, to be a reserved but nevertheless effective platform for international policy coordination.

## SARS experience and health system aspects of the COVID-19 epidemic

In 2003, China/Hong Kong experienced the SARS epidemic in the second quarter, which reduced output in the third quarter considerably in Hong Kong as well as in parts of mainland China. This incident has motivated several researchers to look into the macroeconomic effects of an epidemic where production losses due to the illness of workers/managers and death among the workforce were one key element of analysis. The SARS epidemic was over relatively fast and did not become a major shock to the world economy; not least since China at the time represented only 4% of world GDP. Döhrn (2020) has estimated that China’s decline of real income was 2.4% in the first quarter of 2020. This negative income effect will negatively affect the US, the Eurozone, the UK and other countries. The international diffusion of COVID-19 may be expected to be large: Those infected with the respiratory disease SARS could be rather easily identified, while people infected with the novel coronavirus often show no visible symptoms of the disease.

**Fig. 1:**
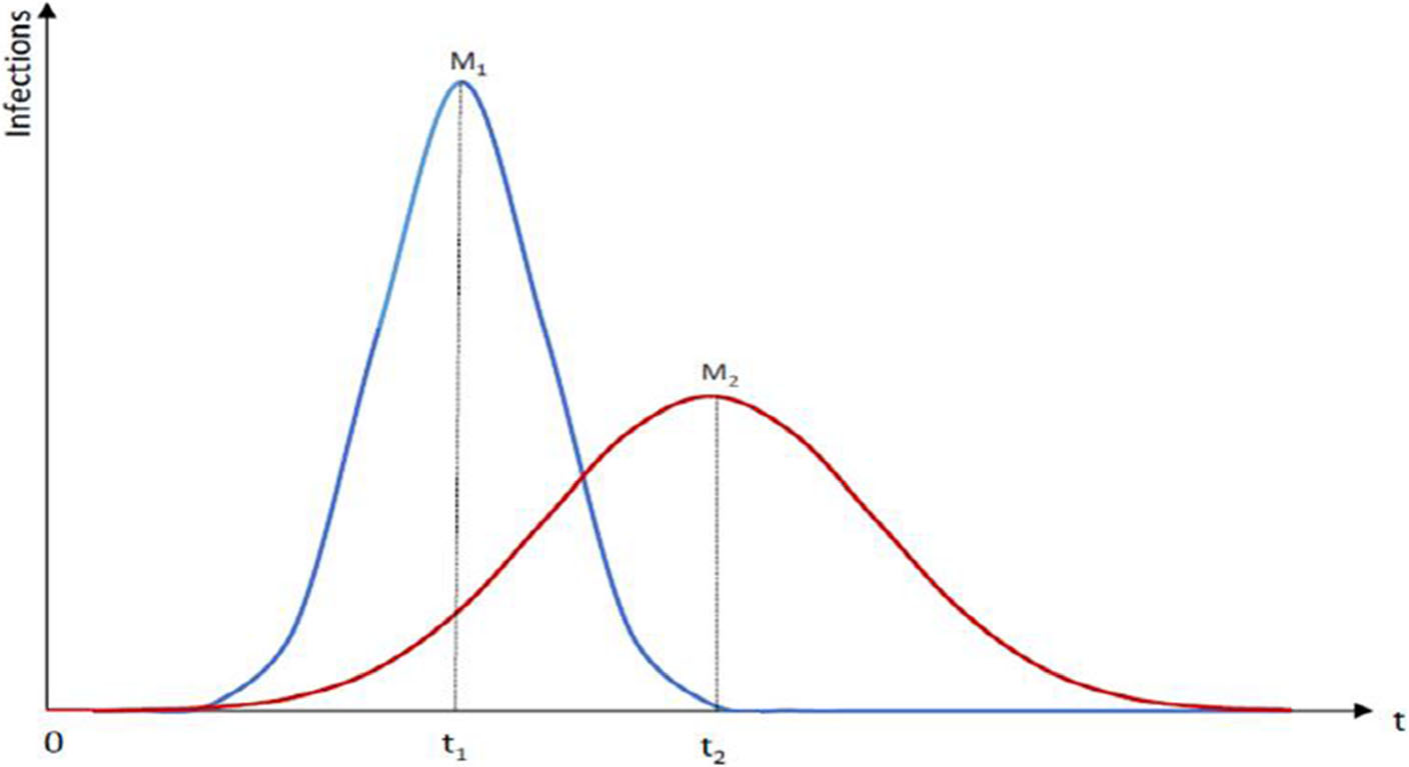
The Effect of Health Policy Intervention on the Peak of an Epidemic. Source: Own representation

One key challenge with an epidemic concerns the burden for the health system and hospitals, respectively. If the personnel in the health system and the capacities of hospitals approach critical limits rather quickly in an epidemic setting, the mortality figures will rise quickly. From this perspective, it is quite important in every epidemic scenario that health policy measures help to postpone the peak of infections and thereby to bring down the level of stress on hospitals to a manageable level. Ideally, health policy shifts the peak from M_1_ to M_2_ on the time axis and in this context adequate testing and broad quarantines are often crucial (see following Fig. 1).

It is also noteworthy that of those infected with COVID-19, circa 80% of patients recover with relatively mild symptoms, while 14% have serious illness-related complications and about 6% face critical illness (ECDC 2020). The latter effect can be expected particularly in the age group above 65. From this perspective, the median age of countries is of interest: The higher the median age in the respective society is, and the higher the share of elderly people in a country considered, the higher the risk of a high morbidity. From the perspective of morbidity, Japan, Germany, Italy, Spain with a rather high share of elderly people could face more problems than, for example, France, the UK or the US (see following Table 3).

While it is clear that an epidemic could to some extent contribute to a rise of the overall health expenditure-GDP ratio as the number of inpatients in hospitals is rising – and output will transitorily fall – one cannot rule out that this ratio is almost constant (or could even fall); the latter case occurs if the incidence of morbidity is rather high for groups with underlying conditions and in the older age brackets so that elderly patients infected with COVID-19 could die rather suddenly and will not experience the normal two last years of life. In these last two years, in the United States, 30% of life time health expenditures occur (DPE 2016). If infected elderly patients die one year earlier than expected, the health expenditure during the last two years of life of the elderly affected would be cut by about half. Only broad statistical analysis in the future will shed more light on these aspects.

As regards the length of the time period of the infection, it is clear that China was the starting point and might return to full production by mid-2020, but the geographic spreading of the epidemic – with a certain number of patients travelling from various countries for business or tourist or family trips to China – will see time lags in the eruption and development of the epidemic. It seems that Portugal, for example, was affected rather late in Europe and also some countries in Latin America could be affected with a considerable delay so that the infection peak in parts of Europa and Latin America could be at least a quarter after the peak in China. As regards the diffusion of the epidemic in the EU – outside Italy – it is possible that travelling between Italy and several neighboring countries (and between China and EU countries, including Italy) has brought a critical number of imported infections which will not make it possible to easily control the epidemic. However, as the SARS epidemic has shown, a single infected tourist coming to Italy could have created the critical number of infected people in Italy. More research is needed to identify the sources of the epidemic in Italy. In the general public discussion, many people are likely to consider it plausible that immigrants are to be blamed – hence the epidemic could become a driver for populist debates in Europe or the US (and elsewhere).

It is not clear whether or not in autumn 2020/winter 2021 a second wave of the pandemic could start. Hence, it is still not clear whether there will be only a short-term one-off negative economic effect in most OECD countries and China as well as other countries; in this context both the financial sector and the real economy could be affected. In some cases, infections might also concern members of parliament or governments in various countries which in turn could made political decision-making more complex. The legal system of the countries concerned might in turn face a wave of liability and litigation cases in the context of epidemic with many novel legal questions faced in many countries.

As regards forecasts of international epidemic incidence, a group of researchers at the Johns Hopkins University has suggested an interesting approach which is mainly based on international and national air traffic passenger links (Gardner et al. 2020). According to this forecast, from late January 2020 the United States would be expected to be a country – with many air traffic links to China – that could face a serious challenge in the context of the epidemic. It is not fully clear whether or not the Trump Administration has considered this research and its implications. In talks with the leading insurance companies on March 10 (and in the days before), the Trump Administration has negotiated that the costs of testing for the novel coronavirus would largely be covered by these companies. However, with 13% of the population without health insurance coverage, there is some specific US health care problem since people who are uninsured might turn to physicians rather late or not at all, if they have symptoms that resemble COVID-19. Illegal immigrants also might become a problem in the fight against the epidemic in the US. An apparent gap in terms of US health management is the lack of testing in February 2020 as pointed out in the international comparison of the US, the Republic of Korea and China (Meyer and Madrigal 2020).

Illness of the workforce as well as death reduce the effective labor input in the macroeconomic production function (and in the production function of individual firms affected by such cases in the respective workforce). Jonung and Roeger (2006), in particular, focused on the effects of a pandemic on tourism and trade as two sectors significantly affected by an epidemic shock abroad – with the potential of an international transmission of the disease; the main insights from this study were that while a pandemic would take a large toll in terms of human suffering, it would not be likely to be a major threat to the EU economy. Typically, output would face a short-term decline but thereafter it would recover rather quickly. A crucial element of an epidemic shock in the first quarter of 2006 – the scenario considered - would be the negative effect on the tourism and entertainment sectors that accounted for 4.4% in the EU25 and also in the US. If one would assume an 80% output decline in demand, the output decline would be 3.5% of GDP in the next quarter and, for the whole year, the aggregate demand effect would translate into a real GDP dampening of 0.5%. However, in the following year, GDP would increase by one percentage point more than in the baseline scenario. Clearly, within the EU, southern countries/countries in the Mediterranean area could be assumed to be particularly affected by the epidemic shock since the share of tourism and entertainment in these countries would be relatively large. In the Jonung and Roeger (2006) approach, about 2/3rd^s^ of the European output shock is supply-induced while 1/3rd is demand induced. The key finding of the authors thus is that a strong output reduction – relative to the business-as-usual case – will occur in an epidemic, but part of the dip in output will be recovered the following year.

At the same time, one might add that Germany would be particularly negatively affected because of its relatively high export-GDP ratio (relative to the country size). An output dampening effect on Italy, Spain, France and Germany would be an economically relevant output dampening effect for the whole of the Eurozone and the EU, respectively. Given the size of the Eurozone, a dampening on Eurozone output would translate into a dampening effect on US exports and output, respectively; and the same applies to a dampening effect on Chinese exports and real GDP. One may also note that the authors did not consider the role of rising costs in the health system. Such costs could indeed be considerable and since health costs in the US, Germany/France (Western Europe; read: Eurozone) and China differ considerably, one should indeed consider the effects of an international epidemic shock on the relative health care costs and the implications for the respective trade balances and current account positions, respectively.

As regards the US and the Eurozone, it is useful to consider some key aspects of the respective health care systems; for simplicity, the Eurozone will be considered here only as the sum of Germany and France – occasionally as Germany, France and Italy. The main differences between the US and Germany are as follows:

- The health care expenditure-GDP ratio is 18% in the USA (for 2018), but only 12% in Germany and France, while life expectancy in the Eurozone is clearly higher and infant mortality lower than in the US (for more see Appendix 6 Fig. 10). Disregarding certain fields of medical excellence in the US, one cannot overlook that the US health system is partly inefficient. It is quite strange that the number of gynecologists per women in the US is only one half that of the corresponding number for Germany. Moreover, an average clinical surgery in the US will cost three times as much as in the US (Göpffarth 2012, p. 30).
- If health care in the US is on average 35% more expensive than health care in Germany/France, there is a serious macroeconomic implication: Assuming that the share of US profits in US gross domestic product is 1/3rd - as is often assumed for western OECD countries – US exporters have a health cost related disadvantage vis-à-vis the Eurozone (Germany/France/Italy/Spain/Netherlands for simplicity) of (2/3^rds^) of 6% = 4%; inefficiencies in the US health system effectively amount to a 4% export tax. Indeed, in the US, health insurance for workers and employees is typically related – outside of Medicare for those aged 65 years and over, and the poor strata which get Medicare from government – to having a job so that the inefficiencies of the US health care system is equivalent to an export tax of the US of 4% (the health care-GDP ratio and the health insurance contribution rate, respectively, will raise the reservation wage in labor markets). The Trump Administration’s debate about an excessive US trade balance deficit thus should start with taking stock of the inefficiencies of the US health care system and – related to this – the apparently enormous lobbying power of part of the US health sector and the lack of price transparency and competition in the hospital sector. By contrast, the health system of Singapore relies on a strict benchmarking of hospitals in Singapore, regardless whether those are private or publicly organized (for an overview of the Singaporean health system in comparison to the US, see US Commerical Service 2015).
- An epidemic affecting all major OECD countries would raise the health care expenditure cost relative to GDP in the US and in the Eurozone, namely through higher expenditures on the one hand and a lower GDP which will reduce due to a rising illness rate of the workforce. If there were to be a full-blown US (or EU) COVID-19 epidemic, hospital costs would increase strongly. As regards the United States, this could mean that the US comparative disadvantage in labor-intensive sectors – effectively also representing high health care costs – would further be reinforced and hence the US trade balance deficit-GDP ratio and the current account deficit-GDP ratio would rise.

Health system reforms can, of course, not be designed and implemented in the short run, but there is no doubt that such reforms should be carefully considered in the EU and even more so in the US. The stress impulse from the COVID-19 epidemic reveals these problems.

It is rather surprising that the enormous US lead in health expenditures relative to GDP – or to life expectancy years – has gone relatively unnoticed over decades in macroeconomic analysis: The US spends 1/3^rd^ more than Western Europe, but has a lower life expectancy and a higher infant mortality rate which is a real puzzle for the US health system and is part of the US weakness in international competitiveness in the production and export of goods, respectively. Within the OECD, every member country could benefit by learning something from every other member country; thus comparative system analysis should remain an important and useful field of International Economics - which it has not been since the end of the Cold War.

**Fig. 2:**
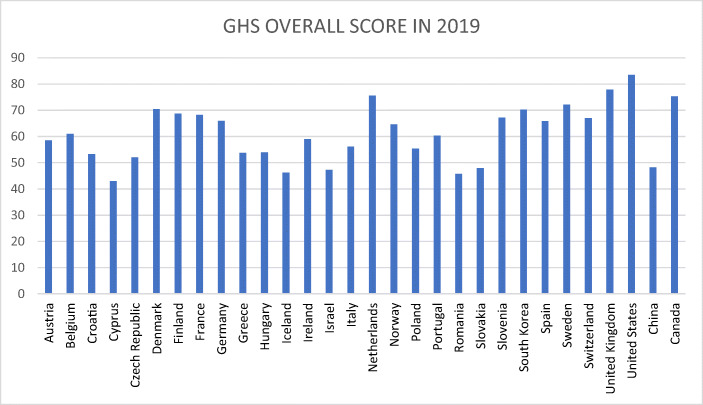
The Global Health Security Index Overall Score, Selected Countries, 2019. Source: Own representation based on data from NTI/Johns Hopkins University (2019)

**Fig. 3:**
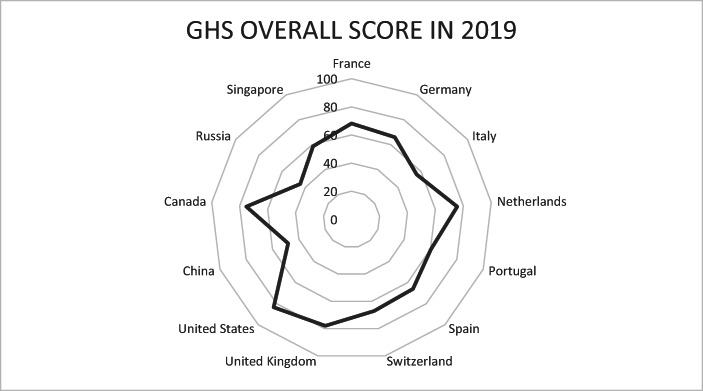
Global Health Security Index, Overall Score, Selected Countries, 2019. Source: Own representation based on data from NTI/Johns Hopkins University (2019)

As regards the findings of the Johns Hopkins University with regard to its global health security indicator (NTI/Johns Hopkins University 2019) about preparedness for dealing epidemics, it is noteworthy that with respect to pillar 4, namely *sufficient & robust health system to treat the sick & protect health workers,* many EU countries have a rather modest ranking and are ranked in the middle group of the 195 countries considered in the Global Health Security Index: Ireland (ranked 41), Luxembourg, Slovakia, Greece, Czech Republic, Italy, Romania, Hungary, Lithuania – and, in the weakest group, Estonia (behind South Africa) which is astonishing and not really acceptable as a status for an EU country. This at least points to the problem that the EU so far has not sufficiently considered a minimum level of health system quality as a requirement for EU membership; indeed including such a quality requirement in the Copenhagen Criteria II (an updated version of the Copenhagen Criteria) should be considered in the medium term by the European Parliament, the Commission and the Council as well as the EU member countries. There is considerable variation amongst OECD countries, plus China and Singapore (see following Figs. 2 and 3). Explaining the GHS index position of individual countries is an interesting question.

As regards the Global Health Security Index, it is remarkable that Russia, China, Italy and Spain have rather weak positions in the aggregated overall index as the above graph shows. However, even some high per capita income countries – Singapore and Switzerland – are not showing a strong position. Taking a look at the number of infections in early March 2020, China, Iran and Italy were leading countries, in terms of mortality the US had a much higher ranking than in the comparative number of infections; this points to a large number of non-identified infections in the United States. Weak points in the US health system could explain this.

It is clear that an international epidemic poses serious risks to the world economy. The ability for firms in all countries to rely rationally on an international division of labor and knowledge is clearly undermined and put at risk if a large number of countries do not achieve high quality indicator marks in the Global Health Security Index.

## Cross country regression results with the GHS Indicator and trade and FDI intensity

As regards the quality of the health care system, it is important to understand the link between the quality indicator Global Health Security index and real per capita income. The relevance of the Global Health Security Index for economic analysis is crucial in two ways:

- The index presents the respective country’s position in a key field of health care
- The index could be used as a health system-related proxy for the effective labor input available in production, possibly including foreign experts and managers flying into the country in order to provide certain services for the production of goods and services which are not fully covered in the statistics, but which play a key role for subsidiaries producing abroad: The higher the ranking in the GHS, the higher the willingness of such experts and managers to temporarily work in the country concerned and to the extent that the GHS index is a proxy for the quality of the health system, one may also assume that the effective use of the workforce could be reflected here (more healthy workers contributing to value-added). Other variables which could explain per capita GDP could be considered in a cross-country regression and the results are straightforward as shown subsequently.

A useful descriptive analysis is to focus on a scatter plot of the per capita GDP figures and the Global Health Security index which shows that both data series are positively correlated (see subsequent figure). The reasons for such correlation have to be identified: Higher per capita income will generally go along with a higher demand for health care services, and better health – based on a better quality of the health system (as proxied by the GHS index) – should contribute in various ways to a higher per capita real GDP (e.g. positive supply-side effects of better health on potential output). The sub-indices with a particular focus on the epidemic absorption quality of the respective health system could also be analyzed for specific research interests.

**Fig. 4:**
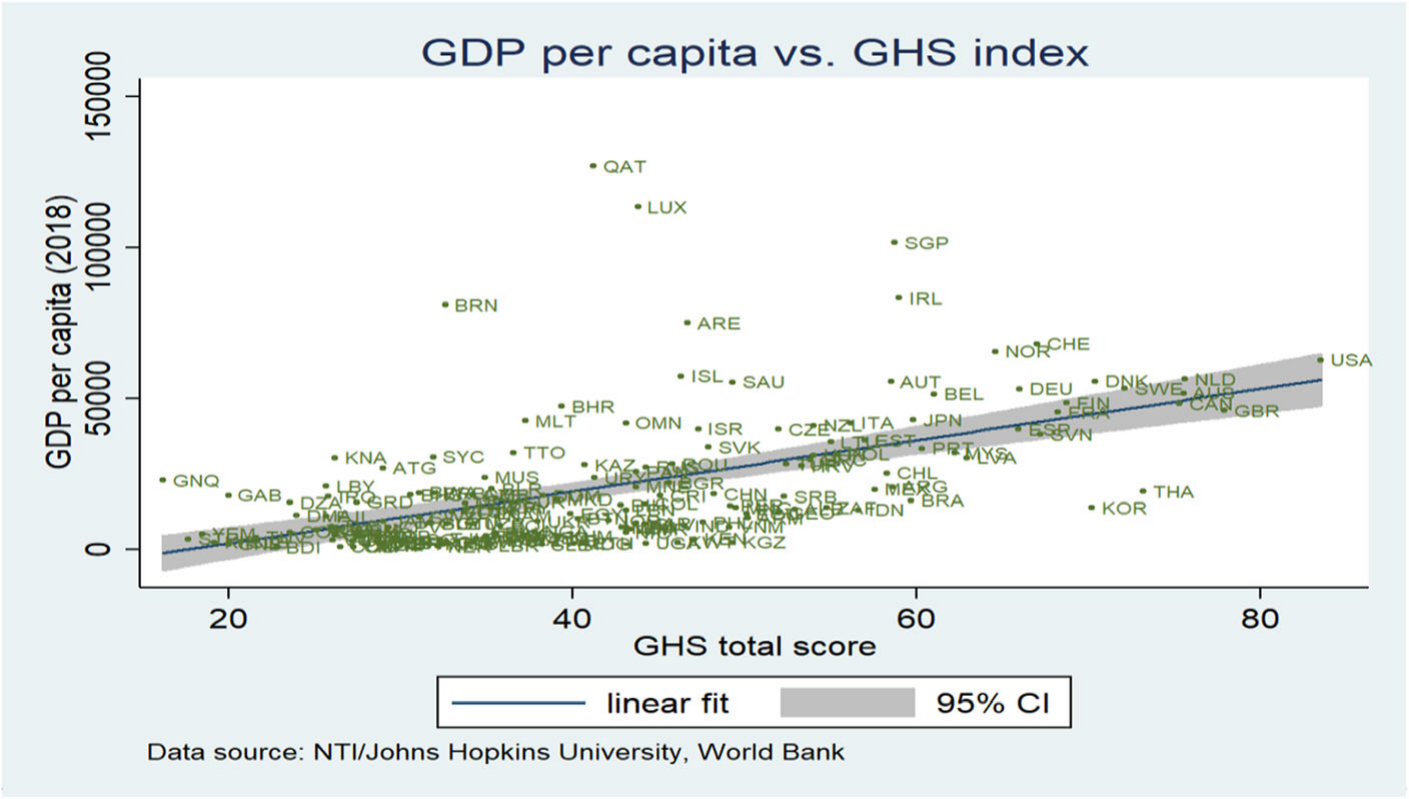
Scatter Diagram for Per Capita GDP Figs. (2018; PPP) and Global Health Security Index (2018)*.* Source: Own representation using data from the NTI/Johns Hopkins Global Health Security Index and World Bank

**Fig. 5:**
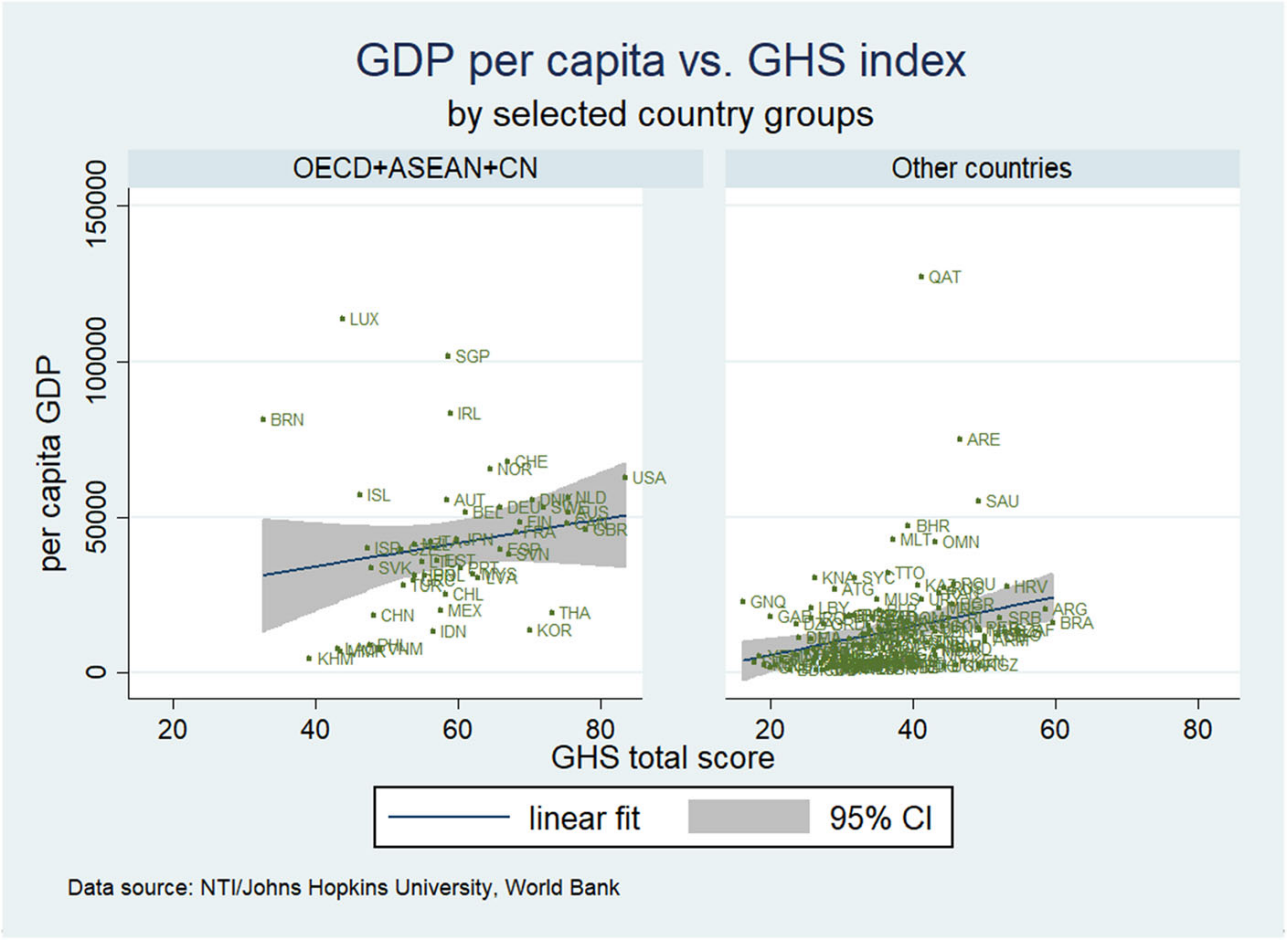
Scatter Diagram for Per Capita GDP Figs. (2018; PPP) and Global Health Security Index (2018) for High Income Group (OECD, ASEAN and China) and Other Countries*.* Source: Own representation using data from the NTI/Johns Hopkins Global Health Security Index and World Bank

**Fig. 6:**
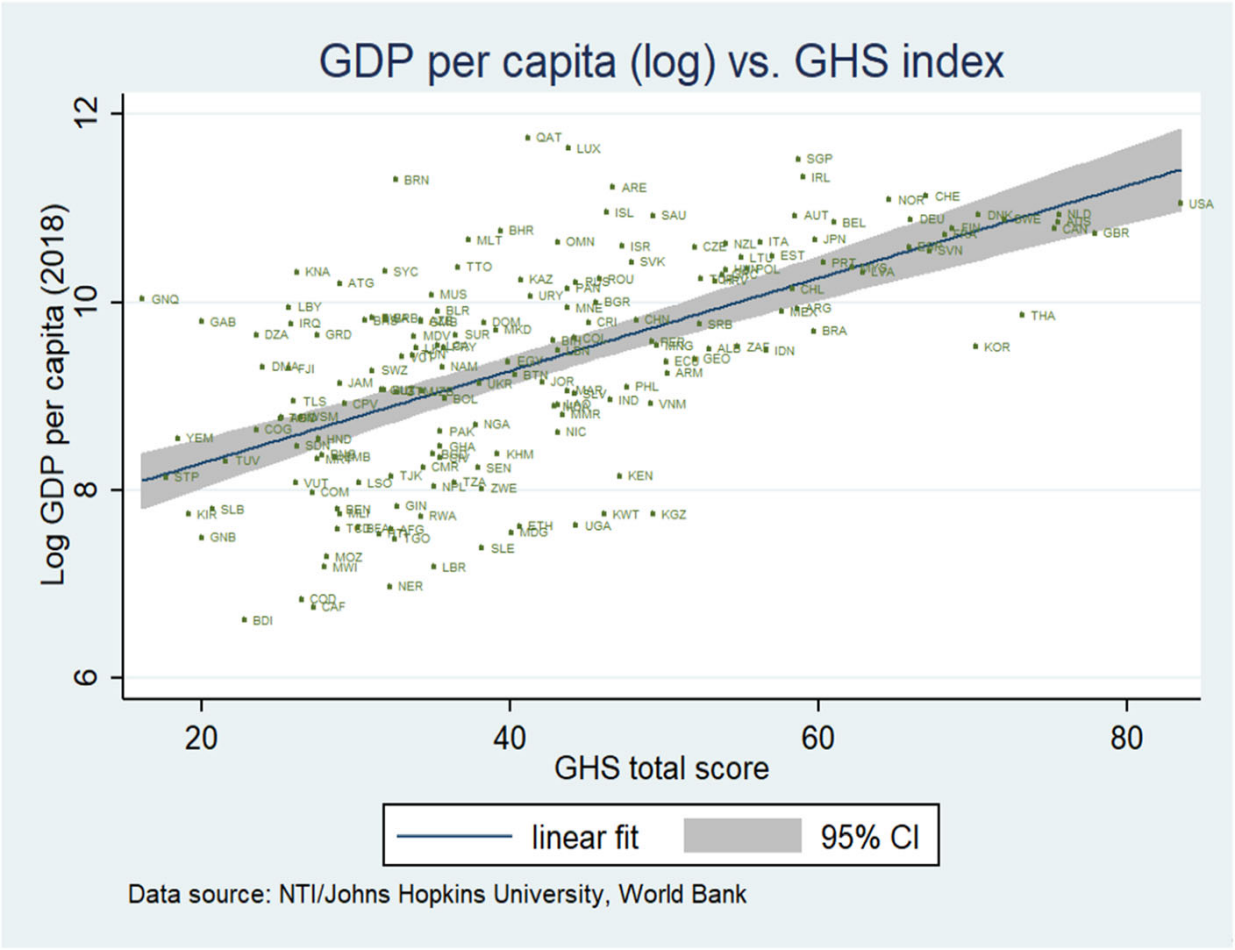
Scatter Diagram for Log GDP Per Capita Figs. (2018; PPP) and Global Health Security Index (2018)*.* Source: Own representation using data from the NTI/Johns Hopkins Global Health Security Index and World Bank

Subsequently, at first (Figs. 4, 5, and 6) a scatter plot for a linear relation of real GDP per capita (PPP) and the GHS index (total score) for all 161 countries is considered: The US is below the average relationship for all countries; here, Luxemburg and Qatar may be considered to be clear outliers. One could also consider the relationship between the log of the GDP per capita and the GHS index, the idea could be to take into account implicitly that real per capita income and health stands for a non-linear relationship (assuming certain linear properties of the construction of the GHS index). Relative to per capita income, the United States and the United Kingdom – and even more so the Republic of Korea, Thailand and Brazil - stand for an above-average GHS index position, while Germany and Japan, for example, have a below-average GHS score. We get an interesting finding when the overall group of countries are split into a high income group – defined as OECD plus ASEAN plus China – and a low income group (“other countries” in the subsequent figures). With this sample split, we can see that quality of the US health system position again is below that of the high income group average relationship; while Brazil is better than the average relationship would suggest. More interestingly, the high income group shows a much higher per capita level than the poor countries while the slopes for both groups of countries are rather similar. This raises the question of whether or not the quality of the health sector itself has an impact on per capita income which itself raises complex issues. Nevertheless, it is interesting to consider a rather simple regression subsequently which will focus on explaining per capita income through key economic variables, including trade intensity (implicitly standing for specialization gains and higher innovation intensity associated with a more open economy).

A simple cross country regression for explaining per capita real GDP (purchasing power parity figures) through the GHS index, the true trade openness and the foreign direct intensity – for 174 countries – shows a good regression fit for this simple approach based on 2018 figures; true openness is a measure for trade intensity corrected for the size of the economy (small countries, proxied here by GDP relative to the average GDP in the sample of countries) and the variable thus reflects both the international division of labor and effective import competition. The true FDI intensity (FDI inflows and FDI outflows) is a similar variable for foreign direct investment; in addition, the true FDI inflow intensity was included. However, neither of the FDI variables were significant.

The regression (Table 4), which included the true FDI inflow variable, explains 44.6% of the variation of real per capita GDP across countries and both the GHS index and the true trade openness variable are significant at the 1% level. The coefficient of the true trade openness is about three times as big as that of the GHS indicator. If one takes logs of the real per capita GDP figure, the coefficients are better to interpret, namely as a semi-elasticity and the adjusted R^2^ rises slightly to 46.7; note that in this variant, the true inward FDI variable was dropped and only the true FDI intensity is used. Once longer time series for the GHS index would be available, panel data analysis with this indicator will become possible so that one could shed more light on the link between health system quality and economic welfare.

An important policy conclusion to be drawn here is that the IMF, the OECD, the EU, the World Bank and other institutions, which try to support economic growth in the world economy through specific programs for member countries or partner countries, should pay more attention to the quality of the health system of the recipient countries.

### US and EU health system problems

As regards the US, one should also not overlook that the case of a serious illness in the family is the most important risk factor for a middle class family to fall from this position into poverty. Case and Deaton (2020) have analyzed the problem of death from despair/suicides - and the issue of the opioid crisis - in the US and have shown that in Western Europe, only Scotland has a suicide rate that is similar to the high rate in the US. The share of uninsured Americans has reduced under the Obama Administration, but under the Trump Administration it has increased from a share of 11% to 13% in 2019.

One may point out that personal bankruptcy due to health care expenditures is a rarely known phenomenon in EU countries with a general health insurance coverage, while that type of bankruptcy is a frequently occurring problem in the US and many households face the problem of living in fear of not being able to pay the next health bill. A survey in the US found that medical bills are the most important cause of consumer bankruptcy – with 18–25% directly caused by medical debt cases (Austin 2014). In another survey, a key finding was that 56 million Americans under the age of 65 had trouble paying medical bills in 2013 (Lamontage 2014). 10 million American adults are expected to face problems with paying medical bills although they have year-round insurance (Lamontage 2014). For discussion of further aspects of health related to productivity aspects in the US, see DPE (2016).The COVID-19 epidemic is likely to create many additional health problems in the US; some patients might die, but most will, of course, survive – however, in many cases, high medical bills could be faced. In the US, the epidemic therefore could likely become an impulse for a decline of real per capita consumption, more so than in the Eurozone or the EU, respectively, where universal health coverage is common. One also has to anticipate that many people in the US will not undergo early testing for the coronavirus since they are afraid of high medical bills and this, in turn, will bring a higher mortality rate in the US than in the Eurozone. In the US, this could lead to a delayed epidemic which would lead to much higher health and output costs in the US than a European-type of broad health insurance system would imply. However, one may point out that a simple extension of Medicare options to individuals below 65 years of age will not solve the US health system inefficiencies alone; the question of how to organize more competition and incentives for healthier living (e.g. without the problem of obesity) as well as lobbying aspects would have to be considered. Econometric work that looks only at US health expenditures and not at the ratio of health expenditure to national income (or GDP) is often misleading for policy reforms.

As regards the ability of health systems to cope with the coronavirus epidemic, the availability of intensive care beds in hospitals will be crucial; assuming that particularly the elderly population faces a high risk in this pandemic, the ratio of acute care beds to the population above 65 years of age would be of particular interest. As regards these figures, the subsequent table gives information for OECD countries. At the beginning of the COVID-19 challenge, there is, of course, a certain capacity utilization of intensive care beds which differs across countries – the higher this capacity ratio is, the more difficult it will be for the relevant health system to accommodate the coronavirus shock. One can see, particularly with relation to the numbers of acute hospital beds, a large variation of capacity across countries – Japan and Korea which have coped relatively well with the COVID-19 epidemics in their countries were better equipped to cope (7.79 and 7.14 acute beds per 1000) than Italy (2.62), whereby the United Kingdom and United States also have relatively low numbers of acute care beds per 1000 population (see following table). For data on acute care beds per 1000 population over 65 (higher risk age group for COVID-19), see Table 6. Here again significant variation is apparent, from 51.92 in Korea to just 10.39 in Sweden. Within the EU, some member states have greater capacities than others relative to the older population, namely Slovakia (32.78), Austria (29.50), Poland (28.26) and Lithuania (28.13). Italy with just 11.74 is towards the bottom of the table. Here some other countries which are seriously affected by the coronavirus come under pressure, namely Spain, Denmark, the Netherlands and the United States.

**Table 6:** Number of Acute Care Hospital Beds per 1000 Population Aged 65 and Over for Selected Countries

Country	Acute Beds per 1000 Over 65
Korea	51.92
Slovak Republic	32.78
Austria	29.50
Poland	28.26
Lithuania	28.13
Japan	28.08
Germany	27.80
Belgium	27.36
Hungary	22.87
Slovenia	22.31
Czech Republic	21.89
Ireland	20.64
Chile	20.07
Switzerland	19.85
Israel	19.24
New Zealand	18.44
Iceland	18.15
Estonia	17.85
Greece	16.67
Latvia	16.52
France	16.07
United States	15.97
Netherlands	15.79
Denmark	13.56
Finland	13.36
Spain	12.81
Italy	11.74
United Kingdom	11.73
Canada	11.62
Sweden	10.39

Source: Own calculations and representation of data from OECDStat, Health Care Resources, data for 2017 or latest available year and Statistisches Bundesamt for Germany; UK: national statistics

The quality of the health care system could also be relevant for the attractiveness of a country as an investment location. Top managers and international investors certainly will be interested in a high quality of health system in the host country locations envisaged. From this perspective, one could plug the NTI/Johns Hopkins Global Health Security Index findings (once more data points are available) into a modified modern gravity equation for foreign direct investment – for a basic FDI gravity modelling analysis, see Welfens and Baier (2018).

## Theoretical macro aspects of the coronavirus epidemic

The role of health system costs has already been mentioned above and one may point out that this problem has rarely been considered in the literature. In aging societies, health care cost will tend to increase further over time. As regards aging, the speed of aging in Germany, Spain and Italy exceeds that of France after 2025 considerably. From a political economy perspective, one cannot exclude that important social and political conflicts will run across age brackets. In the case of BREXIT, for example, the majority for BREXIT in the referendum in 2016 (and in the UK General Election of December 2019 in which there was a majority for pro-BREXIT parties) was in all age groups above 45 years; incidentally, this suggests stronger EU disintegration dynamics after 2025 in part of the European Union.

As regards the effects of an epidemic on the overall economy – with a tradables sector (T) and a non-tradables sector (N) – a simple analytical starting point is the Mundell Structural Equilibrium Model which looks at the equilibrium conditions in the T market and the N market as well as the money market (money market equilibrium is portrayed in the MM curve:

1 $$ M= Pm\left({Y}_0,{i}_0\right) $$

where M is the nominal money supply, P the aggregate price level consisting of a tradables price sub-index P^T^ and a non-tradables price sub-index P^N^; Y is output, i the nominal interest rate, m is the real demand for money). The MM curve in P^N^-P^T^ space is negatively sloped as P = (P^N^)^b”^(P^T^)^1-b”^ where 0 < b” < 1 is a weighting factor. The TT curve – portraying T-market equilibrium – is positively sloped as is the NN curve, portraying N market equilibrium. The condition for T market equilibrium in Mundell’s structural non-tradables/tradables model can be written with a minor modification of the original model as (A^T^ is the knowledge of technology in the T-sector, K is the overall capital stock, L the labor force and hL is the effective overall labor force in the economy where h is a positive parameter which indicates the average health of workers; again, the health insurance coverage ratio could be a good proxy for h. A’ is real wealth of the private sector which enters the demand equations):

2 $$ {T}^s\left({P}^N,{P}^T,{A}^T,K, hL\right)={T}^d\left({P}^N,{P}^T,A'\right) $$

A^T^, K and L are given, h is a policy parameter here; the higher P^T^/P^N^ is, the higher the share of the overall capital stock K and the overall labor force, L, employed in the T sector. A^T^ positively depends on imported intermediate technology-intensive products so that distortions in international logistics amount to a supply shock in the tradables sector. T^d^ is the demand for tradables which negatively depends on P^T^, positively on P^N^ (T-goods and N-goods are substitutes) and the net wealth A’ of the private sector. Similarly, one can state a condition for equilibrium in the N-market (with G standing for real government expenditures that are assumed to fall on N goods):

3 $$ {N}^s\left({P}^N,{P}^T,{A}^N,K, hL\right)={N}^d\left({P}^N,{P}^T,A',G\right) $$

The supply in the N-sector is a positive function of P^N^ and A^N^, K and hL; and a negative function of P^T^. Demand in the N-sector is a positive function of P^T^ and A’; and a negative function of P^N^. In a nutshell, private sector wealth is A’ = M/P + (P′/P)K where the stock market price P′ may be considered to be determined for non-US countries by the US stock market price index P′*; and P′ = eP’* may be assumed here for a small open economy in Europe or Asia. A coronavirus pandemic may be interpreted as a fall of A^T^ and a decline of A’ as P′* will fall - the pandemic will reduce the US stock market price index P′*. The national policy mix then could focus on monetary policy (e.g., a rise of M) or an expansionary fiscal policy or health system related measures to counterbalance the negative coronavirus shock on h. One may assume that the supply shock in the tradables sector has been relatively strong in the first half of March 2020 when production networks were distorted in the context of missing intermediate inputs from China (the epidemic in China still indirectly enforced firm shutdowns in the Chinese export sector until mid-March), but later, in the second half of March 2020, when government regulations in the EU countries closed the entertainment sector there was also a negative supply shock (a decline in A^N^) in the non-tradables sector. To some extent, one could argue that the lockdown of the population in many EU countries and international travel restrictions simultaneously amounted a negative demand shock in the N-sector of these and other countries as well.

With international arbitrage in the goods market and free trade, P^T^ = eP^T*^ (e is the exchange rate, P^T*^ the world market price of tradables; * denotes foreign variables). Hence in a fixed exchange rate regime, changes in P^T*^ will raise P^T^ and this in turn will translate into an excess demand in the money market – firms in the small open economy will export more to eliminate this excess demand so that the MM curve will be shifted to the right through the intervention in the money market, read: The rise of money supply). To the right of the TT curve there is an excess supply in the T-market which, in the case of a small open economy, means a corresponding trade balance surplus. While the original Mundell model (Mundell 1968) assumes a fixed exchange rate, one may, of course, consider the basic model under flexible exchange rates as well. Free trade plus arbitrage will bring about P^T^ = eP^T*^ where in the short run the nominal exchange rate would be determined from the Branson model or interest parity if the setting is one of flexible exchange rates.

To the extent that the COVID-19 pandemic reduces global demand for oil and gas, the tradables world price index (P^T^*) is reduced: An exogenous international price shock from the perspective of a small open economy. In a setting with flexible exchange rates, the short-term reaction of the exchange rate is influenced by US monetary policy – relative to monetary policy in the Eurozone, the UK, Switzerland and China. As US monetary policy has reduced the interest rate by 50 basis points in late February 2020, the US$ should face a temporary depreciation; the small open economy considered here (say, the UK) would therefore face an appreciation of the currency in the setting with flexible exchange rates where the combination of the change of P^T*^ and e should amount to a fall of P^T^. Only in the case that other countries (i.e. not the US) reduce the interest rate rather strongly should one witness a depreciation of the currency so that e rises and the combination of the rise of the exchange rate and the fall of P^T*^ translates into a fall of P^T^ or an increase of P^T^.

**Fig. 7:**
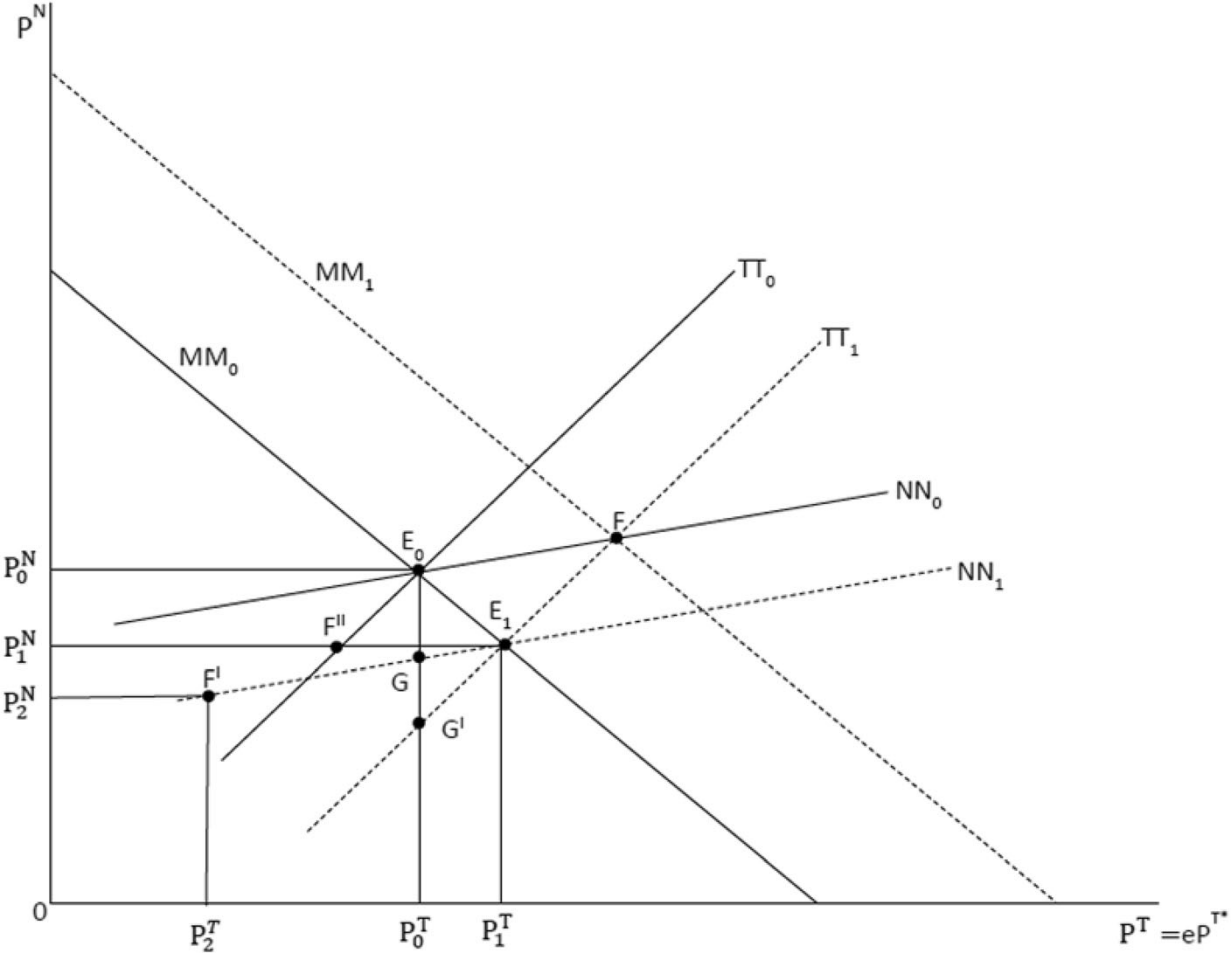
Mundell Structural Model with Tradables and Non-Tradables. Source: Own representation

The slope of the TT curve is a steeper than that of the NN curve since own price elasticity is assumed to exceed the cross price elasticity. The supply in the T market positively depends on the T price and negatively on the N price as well as some supply shift variable V^T^, while the T demand is a negative function of the T price and a positive function of the N price (and a similar economic logic applies, of course, to the N market). The subsequent graph (Fig. 7) is a modified version of Mundell’s book Monetary Theory (Mundell 1968, Chapter 9) where Mundell assumes a fixed exchange rate (e) and full employment (output Y is the weighted sum of N-output and T-output, but one may relax this assumptions).

The supply shock to the tradables sector shifts the T-curve downwards (from TT_0_ to TT_1_), the negative demand shock to the non-tradables sector shifts the N-curve downwards (from NN_0_ to NN_1_). If the new intersection point (E_1_) is on the initial money market equilibrium curve MM_0_, the finding is:

- The price of the non-tradables has reduced in absolute and relative terms
- The price of the tradables has increased

If, however, output is declining, the money market equilibrium curve shifts to the right and the point E_1_ would stand for an excess supply in the money market. One may assume that primarily the price of non-tradables will fall if there is sufficient wage and price flexibility in that sector. The COVID-19 shock in the world economy could bring about an exogenous decline of the world tradables price index P^T*^ and hence in the P^T^ sub-price index so that – with the nominal exchange rate given – the domestic tradables price index will fall. Such a deflationary price impulse for both the non-tradable and the tradable sectors implies that the demand for money will fall as the aggregate price level declines and hence the MM curve shifts further to the right and the excess supply in the money market will increase further; this, in turn, could cause enhanced deflationary pressure which in the medium term would imply declining output.

If there is a deflationary pressure on prices of tradables in world markets, the economy might switch to point F′ on the NN_1_-equilibrium line and hence P^T^_2_ and P^N^_2_, respectively. In the tradables sector, there would be excess demand so that over time there will be a rising foreign indebtedness. At the same time, in a system of fixed exchange rates the central bank would have to intervene in the foreign exchange market and would sell foreign reserves so that the money supply would reduce – the MM curve would shift from MM_0_ to the left (not shown in the diagram). If there is a system of flexible exchange rates, one would have to consider the effects in the short-term BRANSON model, namely that in e-i-space the equilibrium line for foreign bonds would shift upwards so that a nominal depreciation and a rise of the interest rate will occur. The nominal depreciation means that in the Mundell Structural Model, the price P^T^ will increase due to the rise of e; point G’ could correspond to this situation which stands for an excess demand in the non-tradables market.

The original Mundell model implicitly was just N^s^ = N^s^(P^T^, P^N^) and N^d^ = N^d^(P^T^, P^N^, G_0_) where G is the fiscal policy variable (and a similar equation for the tradables sector; plus the equation for monetary equilibrium). An expansionary fiscal policy thus would shift the NN curve upwards. Implicitly, one could also consider a real wealth effect in both the demand for T-goods and for N-goods where real wealth simply would be A’:= M/P + KP’/P where P′ is the stock market price index and K the capital stock. In such a modified setting, one could then analyze both monetary policy and fiscal policy.

If there is a fall of the aggregate price level, the MM curve will shift to the right so that in the previous equilibria E_0_ and E_1_ there would be an excess supply in the money market. An excess supply should normally bring down sectoral sub-price indices and a fall of the aggregate price level; firms would normally also lay off workers. With heterogenous workers, say skilled workers employed in the T-sector and unskilled workers employment in the N-sector, an excess supply of the N-sector would be a problem in the sense that unemployed unskilled workers would need retraining in order to find a new job in the T-sector once that sector rebounds after a major recession.

As regards the macroeconomic order of magnitude of output decline in the context of the coronavirus problem, one may assume a 40% decline of demand in the tourism and entertainment sectors which for the EU, the US and Asia, implies an output decline of about 1.6% in 2020. This means that the projections of about 3% global growth from 2019 are no longer relevant and only about 1.4% global growth should be expected. Output growth could be further dampened if demand decline would be stronger than 40%. However, the dampening could be moderated if an international fall of the relative oil price would occur. Indeed world oil prices have decreased by about $15 per barrel between January 2, 2020, and March 2, 2020 (see Appendix 8 Fig. 11). On March 6, 2020, the Brent oil price stood at $45 per barrel but on March 9 it had already fallen to $31. It should be noted that an output decline of 1.6% in the US and in China would bring a spillover reduction of Eurozone/EU output by about 0.3%. Thus it holds: On top of the direct −1.6% of output growth in the EU, one should get an additional effect which brings an overall output decline of −1.9% compared to a baseline scenario. It will not be easy to adopt a compensating expansionary fiscal policy in EU countries and the EU, respectively. Moreover, it is clear that the shocks to international production networks cannot be healed by national fiscal policy, rather broad cooperation between the EU and Asian countries and the US could be useful here. Given the global excess supply in tradables world markets in 2020, the inflation rate in the Eurozone could fall to 1% in 2020. A decline in the inflation rate in combination with a year long depression in the tourism sector and serious problems in the air transportation and shipping sectors (e.g. cruise ships) could bring liquidity problems for a sizable number of firms – for a small number also solvency problems – so that higher spreads on corporate bonds and reduced loan growth of banks could dampen economic development.

## Health insurance, endogenous time-horizon, life expectancy and economic welfare

In an economic perspective, the role of the time horizon should be considered as linked to the health insurance coverage (h) and the quality of the health care system, respectively. If one lives in a society with a broad health insurance coverage of the population, life expectancy will be higher than in the case of a society with a rather small h. Individuals are typically assumed to maximize lifetime utility (and also typically, for the sake of simplicity, the time horizon is from 0 to infinity which takes away a core problem at the intersection of Economics and Health System analysis. A more realistic and relevant approach here could be to assume that the typical household maximizes utility U which is a function of the quality of life – proxied by h – and the log of consumption C(t) as stated in the subsequent simple approach. The expected lifetime T – relevant for the time horizon over which the maximization of consumption takes place - is a positive function of h; a broader health insurance will bring about a better average quality of life and quality of consumption, respectively. This aspects makes the problem setting more realistic and also more difficult to analyze (households consider here discounted “effective consumption” which is assumed to be represented by h lnC(t) e’^-rt^ (see eq. (4); e’ is the Euler number, r the real interest rate). The setting is rather compact if h is assumed to be constant over time (see eq. (5)); in a broader approach, one will have to make an explicit distinction between h and individual health quality h”i). Even then, two neighboring countries will have migration – say between Mexico and the US or Eastern Europe plus Africa and Western Europe – because there are cross-country differences in h; the average health status in California/Texas, and possibly the whole US, is higher than in Mexico. Not only is the difference in consumption C between two countries considered relevant, but the variable h could indeed also be considered in international migration analysis. The relevance of h and health quality, respectively, can be pointed out through the subsequent graph.

4 $$ U=\underset{0}{\overset{T(h)}{\int }}h\ln C(t)e{\hbox{'}}^{- rt} dt $$

5 $$ U=h\underset{0}{\overset{T(h)}{\int }}\ln C(t)e{\hbox{'}}^{- rt} dt $$

**Fig. 8:**
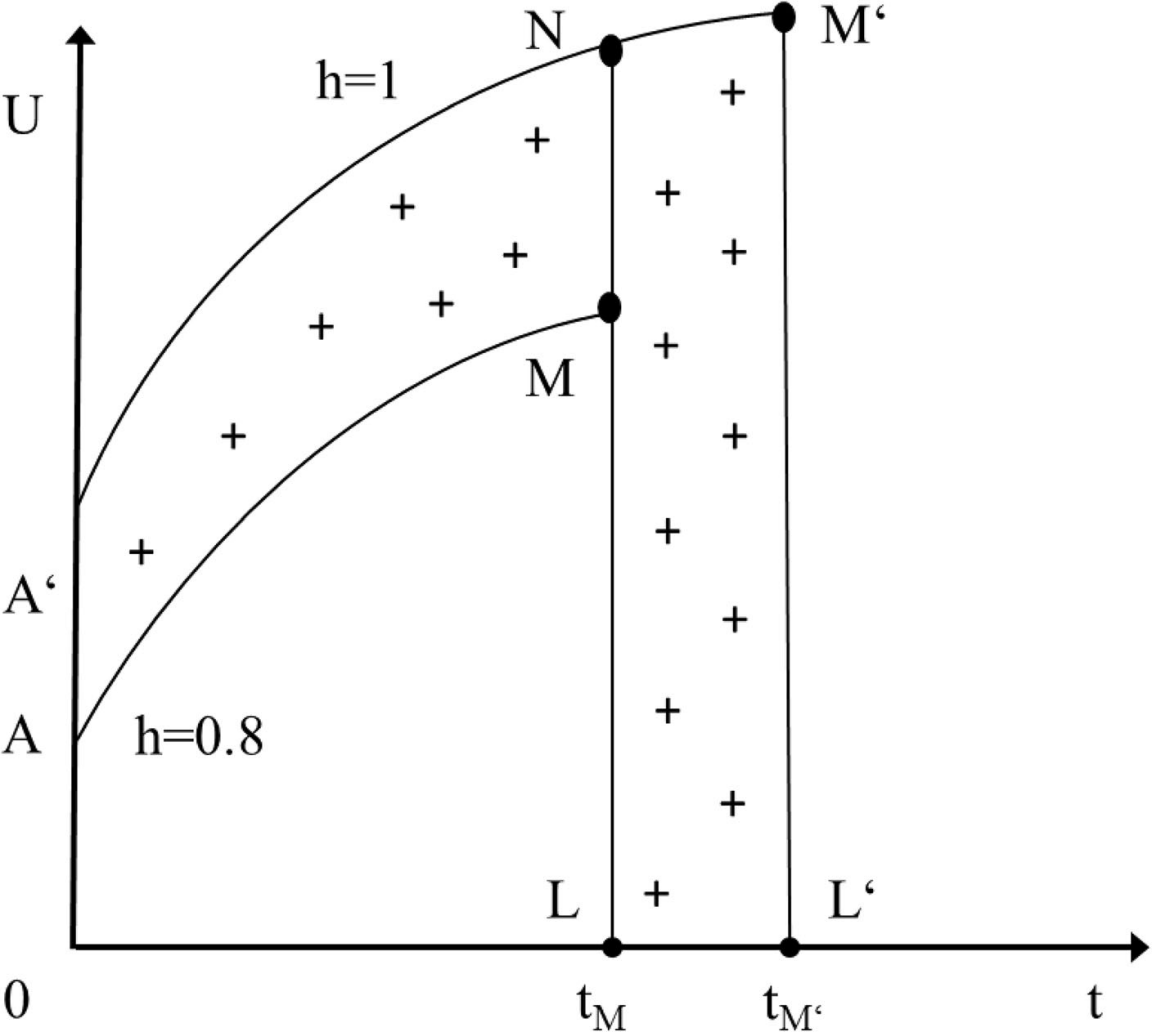
Lifetime Utility Maximization with Endogenous Time Horizon*.* Source: Own representation

**Fig. 10:**
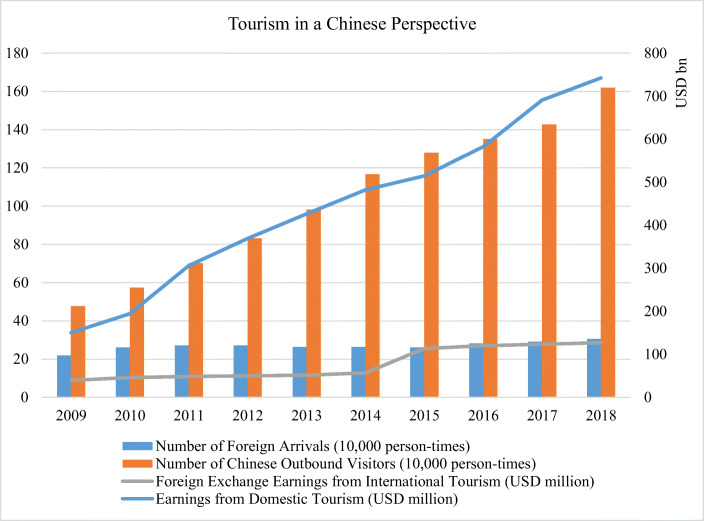
Tourism in a Chinese Perspective*.* Source: Own representation of data from the National Bureau of Statistics of China

The Fig. 8 shows the utility function for the case of h = 1 and a lower value of h, say h = 0.8.

By assumption, the low value of h is associated with a rather short average life expectancy. To put it differently: Raising h from the lower value to a higher value will not only lead to an upward bending/shift of the consumption path, but moreover life expectancy will be raised from t_M_ to t_M’_. The increase in utility through a higher h is shown by the dotted area and it is noteworthy that traditional utility maximization approaches would overlook the role of the welfare gain LNM’L’ Fig. 8.

At the bottom line, the coronavirus analysis presents an interesting analytical challenge in terms of modifying established approaches. Policymakers would be wise to encourage this type of new analysis. Finally, the overlap of health care analysis and macroeconomics seems to be a useful field for further research – the health sector is big enough to affect the overall economy in a significant way and the many effects of Health System reforms on the aggregate demand side (and indeed consumption) and the supply side/the production potential should be carefully analyzed. Macroeconomic policy advisory boards in all countries and international organizations also should consider the new research perspectives.

## Financial market perspectives

COVID-19 is an international epidemic which has raised doubts amongst international investors with respect to US stock market developments and stock market prices in the Eurozone and the UK as well as in Japan, the Republic of Korea and China. The long run rise of relative stock markets came to an end in February 2020; and in mid-March there was a considerable downward pressure on stocks in OECD countries and China (Appendix 11 Fig. 12). Hence, the economic upswing in the US and the EU, plus the UK, has also come to an end in spring 2020. As oil prices have started to fall, this contributes to stabilizing economic developments in the EU and the UK; however, in the US output is dampened in the oil and gas sector and this in turn – along with an anticipated reduction of aggregate demand – will contribute to lower output growth. US monetary policy has reduced the interest rate in early 2020, but its ability to stabilize output growth is quite limited.

One should not rule out that the COVID-19 problem will lead to enhanced political instability in some countries. In a broader perspective, political instability has become an element of certain OECD countries and the Bank for International Settlements’ 2018 Annual Report of indeed has pointed out the problem of political instability and the problem that political instability can easily transform into economic instability (BIS 2018). Political instability could raise risk premiums in the corporate sector and thus dampen investment growth. As regards the impact of a political shock in Europe, the BREXIT shock has been important in recent years for both the UK and the Eurozone. Kadiric and Korus (2019) show that the corporate bond yields have increased in the UK by 23 basis points for maturities of 3–5 years, 21 basis points for 5–7 years, 18 basis points for 7–10 years and for maturities above 10 years by 16 basis points; in the Eurozone, the impact on corporate risk premiums was 9 basis points for maturities 1–3 years, 3–5 years and above 10 years. This means that political shocks could bring about higher corporate risk premiums and hence a dampening effect on investment, output and jobs.

The epidemic is indeed likely to bring liquidity problems for many firms which face certain fixed and variable costs while sales proceeds are falling as demand is shrinking or the production falling due to problems with sourcing of intermediate inputs from the domestic and international economy. Sectoral risk premiums in OECD/G20 countries should indicate part of these liquidity problems on the one hand, on the other hand these problems can be mitigated by economic policy intervention as governments can, for example, allow firms to postpone tax payments or that governments pay quasi-subsidies from broader unemployment insurance funds to firms with reduced capacity utilization (such measures have been announced by the German government on March 13). Government guarantees for bank loans given to solvent firms facing actual and potential liquidity problems could also be useful – in the EU both Germany and France have adopted such measures in mid-March 2020. The basic idea here is to help firms with short-term cash-flow problems. Firms which are insolvent should, of course, not benefit from such government support. If the share of non-performing loans should increase in OECD/G20 countries, one will have to consider government backed increases of banks’ equity-capital ratios. As aggregate demand should pick up in late 2020 and 2021 – partially compensating for the shock-induced demand reduction in the second and third quarter 2020 – most firms should be back on track in 2021. For the Eurozone and the EU, respectively, this could mean that there will be a recession in 2020, but in 2021 there could be a new economic upswing in OECD countries plus China (as well as other countries).

**Fig. 11:**
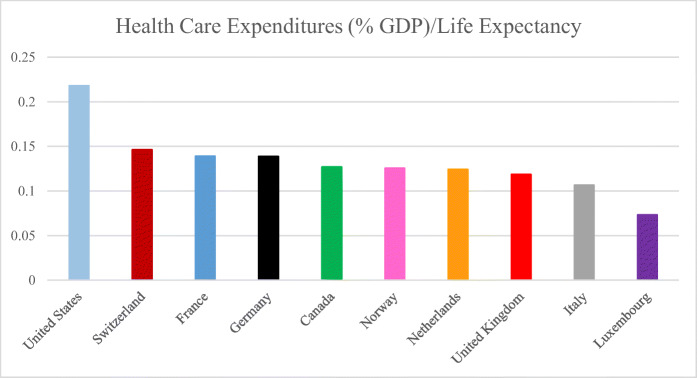
Health Care Expenditures as Percentage of GDP/Life Expectancy for Selected Countries, 2016*.* Source: WELFENS (2019)

**Fig. 12:**
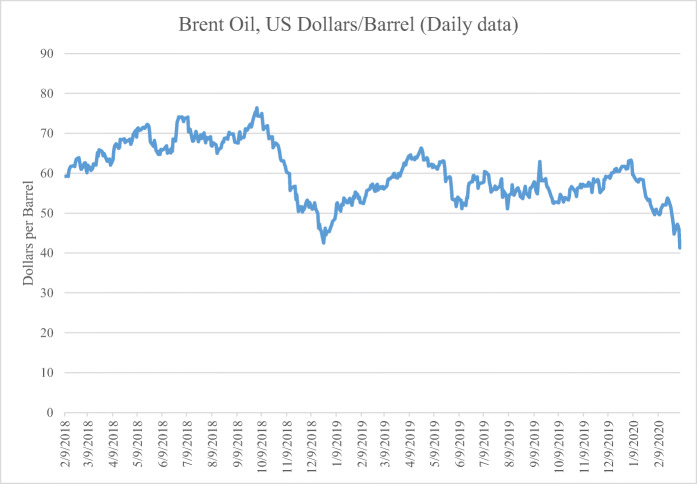
Brent Oil Prices, US Dollars per Barrel (Daily Data), 09.02.2018–06.03.2020*.* Source: Own representation of data available from finanzen.net

As regards oil prices, there could be a considerable decline in the course of 2020 (Appendix 8 Fig. 11), not least since Saudi Arabia und Russia found it difficult to agree on oil production cuts in early March 2020. A strong fall of oil prices will contribute to very low inflation rates in the EU, China and the US, but in the US very low oil prices could also bring about output reduction in the fracking sector and some problems for US banks with large exposure through loans to the oil and gas sector. With declining oil and gas prices there will also be corresponding current account effects in major importing OECD/EU countries while the US could face a worsening of the current account position. Countries that are both net oil importers and have a net expenditure position in tourism should be natural winners from the international coronavirus crisis.

The high volatility of stock market prices and the decline of stock market indices in the western world in mid-March 2020 has generated a broader wave of margin calls so that investors had to provide more equity capital for the banks that had financed stock market investment through loans. Typically, investors would have to sell certain assets including gold whose price fells strongly in mid-March. The gold price might slightly recover as expansionary monetary policy further reduces nominal and real interest rates on government bonds.

## Political economy aspects in the Western world

With respect to the United States, the coronavirus problem is a particular challenge for the Trump Administration which suffers in many fields from a lack of expertise and thus might have serious problems in finding an efficient and effective answer to the questions posed by an epidemic in 2020. It should not be surprising if the coronavirus would have a decisive impact on the outcome of the US presidential elections on November 3, 2020. It is clear for the large majority of the population that government and the political system, respectively, have to take care of the international epidemic – and that the US Congress and the national government/the President of the United States are largely responsible for the policies adopted.

If Donald Trump should not be re-elected on November 3, the European Union would have a reinforced political position since a President on a Democrat ticket would most likely continue traditional US support for regional integration in Europe and other regions of the world economy – which was interrupted under President Trump. This implies that the COVID-19 challenge for the US could have strong political implications in the field of transatlantic economic relations; and the envisaged EU-UK free trade agreement would certainly look different if the EU is in a relatively strong position vis-á-vis the UK compared to the alternative setting where the position of the Johnson government is quite strong due to political backing of an anti-EU oriented newly re-elected Trump. For the EU, there is no reason not to push for a final EU-UK negotiation round in mid-November 2020. Beyond such medium-term perspectives, there are more long run questions raised by the COVID-19 epidemic.

## Growth modelling approach

An epidemic can obviously affect not only cyclical output developments in the medium term but also long run economic development; that is economic growth. Here, a brief look at an adequately modified rather simple neoclassical model setting can be useful. One crucial issue concerns the question of health insurance coverage (h) and its effect on potential output and economic welfare, respectively. In this section, only a few aspects can be analyzed, namely to what extent an epidemic – or waves of epidemics – could affect the long run level of the growth path and the growth rate of per capita income (y) in the steady state (read: In the very long run). For simplicity, a crucial aspect has to be ignored here, namely that typically a higher health insurance coverage will raise life expectancy; an important issue for which western EU countries and the US provide relevant evidence. One may also point out here that a traditional modern growth modeling approach amounts to ignoring this crucial aspect, namely by assuming that households are maximizing utility (depending on per capita consumption C/L) in an approach with an infinite time horizon. This infinite time horizon is a way to simplify some of the task of modelling; however, it amounts to ignoring that the length of a lifetime is an endogenous variable. From this perspective, my emphasis on a systemic comparison of social market economies in Western Europe and the US is rather adequate, namely to calculate effective lifetime per capita consumption or effective lifetime per capita income – and here the figures for Germany and France are indeed equal to the US value (Welfens 2019); but the western European countries have the additional advantage that infant mortality is lower than in the US (one could argue that risk averse individuals/parents would thus have a preference for living in Germany/France). As regards the link between health and growth, there is a rather limited range of contributions, which includes, for example, models which consider environmental shocks/health shocks and growth (Bretschger and Vinogradova 2017, 2019). Economic growth models can also integrate endogenous population growth (Bretschger 2019). The subsequent approach is a rather compact modified Solow model with health insurance and the role of health in terms of effective labor input in an open economy with foreign direct investment (FDI). There is asymmetric FDI, namely only inward FDI. Foreign investors will be assumed to invest part of the profits made in foreign subsidiaries in the host country; ß is the share of profits in GDP in the host country and α*ßY is profits of subsidiaries in country 1. The health coverage ratio (h) is considered to be a proxy for the health status of the representative worker.

In a more long run perspective, a simple modified neoclassical growth model can be useful where the basic approach used here relies on Welfens (2011): Denoting real GDP by Y, the share of foreign ownership in the capital stock of country 1 (home country) by α*, the savings rate of domestic households as s (0 < s < 1) and the savings rate of foreign investors as s’ (0 < s’ < 1), we have for aggregate savings S (with * denoting foreign variables, t” is the income tax rate, the profits of foreign subsidiaries are assumed to be untaxed in country 1):

6 $$ S=s\left(1-t^{\prime\prime}\right)Y\left(1-\alpha \ast \ss \right)+s^{\prime}\alpha \ast \mathit{\ss Y} $$

Note that “domestic savings” is proportionate to disposable national income Z where the latter is defined as Z = Y(1-α*ß); it is assumed that a higher health insurance coverage (h) does not dampen the term domestic savings (if one is to consider this aspect, one could use a modified term s(1 − t”)(1 − v”h)(1 − α*ß))Y where v”h is assumed to be in the interval 0,1; v” is a positive parameter. There would be a dampening effect of v” on the level of the growth path of per capita income y:=Y/L and Y in the steady state. One could also consider that there is an explicit health insurance contribution rate t’ so that households savings is s(1 − t”-t’h)(1 − v”h)(1 − α*ß)Y, moreover foreign subsidiaries could consider h to be an indicator of the locational quality of the host country and therefore the second term could be modified as s’α*ß(1+V’h)Y where V’>0; but such refinements will not be considered in the subsequent compact model).

The share of the workforce covered by health insurance is denoted by h (0 < h < 1), K is the capital stock, A is knowledge and ß and ß’ are positive parameters (with 0 < ß < 1; assumption h^ß’(1-ß)^ > 1 so that a higher insurance coverage rate brings a higher production potential):

7 $$ Y={K}^{\ss }{\left({ALh}^{\ss \prime}\right)}^{1-\ss }={K}^{\ss }{(AL)}^{1-\ss }{h}^{\ss \prime \left(1-\ss \right)} $$

Population growth is assumed to be n (an exogenous parameter) and the growth rate of knowledge (a) is also an exogenous parameter:

Defining k’:= K/(AL) and y’:=Y/(AL) – where AL is labor in efficiency units – we can write (with d’ denoting the capital depreciation rate; steady state is denoted by #):

8 $$ dk^{\prime }/ dt=\left(s\left(1-t^{\prime\prime}\right)\left(1-\alpha \ast \ss \right)+s^{\prime}\alpha \ast \ss \right){h}^{\ss \prime \left(1-\ss \right)}k{\prime}^{\ss}\hbox{--} \left(a+n+d^{\prime}\right)k^{\prime } $$

Hence the steady state solution (on the basis of the equilibrium condition savings equals gross investment dK/dt + d”K) is given by:

9 $$ k^{\prime}\#={\left[\frac{\left(s\left(1-t\prime \prime \right)\left(1-\alpha \ast \ss \right)+s\prime \alpha \ast \ss \right){h}^{\ss '\left(1-\ss \right)}}{\left(a+n+d\prime \right)}\right]}^{\frac{1}{\left(1-\ss \right)}} $$

The steady state condition for k’ is derived from the differential equation above which in turn assumes that savings S equals gross investment dK/dt + d’K, while taking into account the enhanced macroeconomic production function. Assuming that broader health insurance is associated with better health and that health represents an implicit consumption good, this formulation of the enhanced growth model is adequate. If, however, one holds the view that health is rather a kind of investment good, one may note that it would be possible to additionally consider the equilibrium condition for the goods markets as S = dK/dt + d’K + E”h where h is health insurance coverage and E” is the average real per capita expenditure on health while E” = e”Z (Z is real national income; 0 < e” < 1; E” thus is assumed to be proportionate to real gross national income Z. In this case, the numerator expression is more complicated, namely (a + n + d’ + e”h(1-α*ß)), and the solution of the differential equation and relevant conditions in the appendix become much more tedious without gaining significant additional insights for the basic discussion here (this additional aspects lends itself, however, to future research steps). The main interest here is to consider a potential trade-off between the effect of a higher h on the level of the growth path and the growth rate of per capita income in the steady state on the one hand; on the other hand, it is interesting to consider the long-term impact of an epidemic shock which will be considered as affecting both the quasi-exogenous growth rate of knowledge (a) and the quasi-exogenous growth rate of the population (n). Just as the massive impact of the Transatlantic Banking Crisis of 2008/09 brought about a downward permanent shift of the level of the growth path of many OECD countries, the global coronavirus shock could bring about a downward shift of the path of the global growth rate; or – as considered here – potentially also a change in the long run growth rate.

Thus the long run equilibrium condition for Y/(AL) is given by:

10 $$ y^{\prime }={h}^{\ss \prime \left(1-\ss \right)}{\left[\frac{\left(s\left(1-t\prime \prime \right)\left(1-\alpha \ast \ss \right)+s\prime \alpha \ast \ss \right){h}^{\ss \prime \left(1-\ss \right)}}{\left(a+n+d\prime \right)}\right]}^{\frac{\beta }{\left(1-\ss \right)}} $$

Consider the following semi-exogenous growth rate of the population (with n’ being an exogenous parameter, C″ and h” are positive parameters so that the parameter h” dampens the epidemic shock parameter C″ (C could stand for a normal flu epidemic or indeed the coronavirus pandemic) which reflects the coronavirus effect on long run population growth):

11 $$ n=n^{\prime}\left(1\hbox{--} C^{\prime\prime}\left(1-h^{\prime\prime }h\right)\right) $$

For the sake of simplicity, this parsimonious formulation ignores the standard aspect of social security that broader social security coverage – here health insurance coverage (h) – is typically found to dampen the growth rate of the population.

Per capita income in the steady state y:=Y/L thus is given by (with e’ denoting the Euler number and t the time index): A(t)=A _0_e^,at^ where A_0_ is the initial level of A

12 $$ y\#={h}^{\ss \prime \left(1-\ss \right)}{\left[\frac{\left(s\left(1-t\prime \prime \right)\left(1-\alpha \ast \ss \right)+s\prime \alpha \ast \ss \right){h}^{\ss \prime \left(1-\ss \right)}}{\left(a+n\prime \left(1\hbox{--} C\prime \prime \left(1-h\prime \prime h\right)\right)+d'\right)}\right]}^{\frac{\ss }{\left(1-\ss \right)}}{A}_0e\kern0.5em ^{\prime}\kern0.5em \mathrm{at} $$

13 $$ y\#={h}^{\ss \prime }{\left[\frac{\left(s\left(1-t\prime \prime \right)\left(1-\alpha \ast \ss \right)+s\prime \alpha \ast \ss \right)}{\left(a+n\prime \left(1\hbox{--} C\prime \prime \left(1-h\prime \prime h\right)\right)+d\prime \right)}\right]}^{\frac{\ss }{\left(1-\ss \right)}}{A}_0e\kern0.5em ^{\prime}\kern0.5em \mathrm{at} $$

It is conceivable that h has a value which makes it neutral for the level of the growth path – see the term h^ß’^ on the one hand and on the other hand the term (a + n’(1-C″(1-h”h)) + d’)^ß/(1-ß)^ in the numerator. In this special setting, raising the health insurance coverage (assuming 0 < h < 1) has a positive effect on the growth rate in the steady state. This still leaves the question of whether or not there is an optimum health insurance ratio in an economic perspective. The political system can, however, decide to fix h at some adequate ratio or indeed set it equal to unity. The insurance coverage ratio h raises the steady state per capita income y#. The growth rate of long run output (g_Y_) therefore is given by:

14 $$ {g}_Y=a+n^{\prime}\left(1\hbox{--} C^{\prime\prime}\left(1-h^{\prime\prime }h\right)\right) $$

The key aspects of growth here is h affects both the level of the growth path and growth rate in the steady state. The effect of h on the level of the growth path is ambiguous, but a rise of h will raise the growth rate of per capita income in the steady state. The case that the health insurance coverage ratio h is neutral with respect to the level of the growth path is analyzed in Appendix 9; “health insurance coverage” can effectively mean different things in the US and the EU – in any case, the US has a system of predominantly private health insurance (see Appendix 10 Table 12) which seems to put rather limited pressure on the pricing of health services and hospitals. If h has a negative effect on the level of the growth path, a rise of h in the course of health system modernization would mean that the population would face a short-term deterioration of the per capita income, but a long run increase of y. If the parameter setting represents such a setting, the time horizon of voters and policymakers, respectively, could play a crucial role. “Short-termism” in the political system might undermine the prospects for health system modernization in the sense of a broadening of the health insurance coverage ratio.

One might want to consider links between the growth rate of knowledge a and h, for example through a rather simple function (with a’ and a” both standing for a positive parameter):

15 $$ a=a^{\prime }+a^{\prime\prime }h $$

The reason for such a positive link between health insurance coverage and the growth rate of knowledge could be the fact that a high ratio h implies that more children will be able to go to school and to finish higher education studies; in a society with a rather small h, illness of parents would otherwise force young adults to interrupt human capital formation to take care of ill parents. Technically speaking, a’ is here the purely exogenous growth rate of knowledge and a”h thus reflects the positive effects of more human capital formation – this includes that a share of individuals with tertiary education will become researchers - on the creation of new knowledge and the growth rate of knowledge, respectively (see on the knowledge production function and the impact of researchers for the case of EU countries Jungmittag and Welfens 2020). In this special setting, raising the health insurance coverage (assuming 0<h<1) has a positive effect on the growth rate in the steady state. This still leaves the question of whether or not there is an optimum health insurance ratio in an economic perspective. The political system can, however, decide to fix h at some adequate ratio or indeed set it equal to unity. The insurance coverage ratio h raises the steady state per capita income y#.

On the basis of (equation 15), we have:

16 $$ {\mathrm{Y}}^{\#}={\displaystyle \begin{array}{c}\left\{{\mathrm{h}}^{\mathrm{\ss}\hbox{'}}{\left[\left(\mathrm{s}\left(1-\mathrm{t}"\right)\left(1-\upalpha \ast \mathrm{\ss}\right)+\mathrm{s}\hbox{'}\upalpha \ast \mathrm{\ss}\right)/\left(\mathrm{a}+\mathrm{n}\hbox{'}\left(1\hbox{--} \mathrm{C}"\left(1-\mathrm{h}"\mathrm{h}\right)\right)+\mathrm{d}\hbox{'}\right)\right]}^{\mathrm{\ss}/\left(1-\mathrm{\ss}\right)}\right\}\\ {}{\mathrm{A}}_0{\mathrm{L}}_0\mathrm{e}{\hbox{'}}^{\left[\mathrm{a}\hbox{'}+\mathrm{a}\hbox{'}\hbox{'}\mathrm{h}+n\hbox{'}\left(1\hbox{--} \mathrm{C}"\left(1\hbox{--} \mathrm{h}\hbox{'}\mathrm{h}\right)\right)\right]\mathrm{t}}\end{array}} $$

Hence the level of the growth path as well as the trend growth rate of Y depend on h; and here the problem could be that a rise of h could reduce the level of the growth path while h is raising the trend growth rate – even if C” is zero. Thus the role of the time horizon of voters and politicians, respectively, will become crucial. One corona shock element in a simple growth model of an open economy as well as a closed economy could be that intermediate input production networks are damaged by the epidemic and pandemic, respectively: a straightforward interpretation would be to interpret such a weakening of input networks as a rise of the capital depreciation rate which, of course, would reduce the level of the growth path in the steady state. One may also note that the population growth rate could be affected – possibly in a positive way during the few months of a broad or partial lockdown of the economy (and the stay-at-home policy of various governments aiming at quarantine effects to slow down the spread of the virus), but the possibly massive economic shock associated with the pandemic is likely to make would-be parents more skeptical about the prospects of raising children in a friendly, social environment and the pandemic could also cost the lives of young women who would normally be pregnant in future years.

## Implications for policymakers

If there is a recession in 2020/2021, an expansionary fiscal policy would have to be adopted which should include novel complementary measures to introduce digital platforms that could offer better future opportunities to source imported inputs from a more diversified supplier pool. Even some aspects of competition law might have to be reconsidered here, namely that the pooling of intermediate imports from non-EU countries should be facilitated for small and medium-sized firms in the European Union. Notification of such pooling would be adequate.

The role of the health systems in open economies should be studied more carefully. For too long this problem has been largely neglected in International Economics. The role of health for productivity, the size of the effective workforce and for entrepreneurship should be studied and comparative international studies could be useful here.

More cooperation in international health policy – a field hardly existing in some countries – is needed and a more formalized pattern of cooperation could be adequate. The role of the World Health Organization is crucial and this indeed is part and parcel of the valuable multilateral system that the EU should defend at the G20 and beyond. It could be quite useful to create an interdisciplinary research network on Efficient and Innovative Health Systems which could be co-financed by G20 countries, but which would have to leave scientific organizers clear freedom in creating international research networks.

Analytical links between health policy and macroeconomics should be studied in a systematic way; as well as links between macroeconomic dynamics and health. In aging societies, expenditures on health care will rise in OECD countries – and also in China in the long run. There is a body of literature that looks into social security, employment, growth and budget deficit dynamics, but there are neglected fields in terms of health system analysis and modern macroeconomics. Moreover, little research has been conducted on the political economy of health care reform. Aging societies in democracies might face particular conflicts in government funding, namely with regard to the extent to which government transfers in favor of broadening health care or broadening pension systems should be designed. Whether or not there is a structural conflict of interests between the younger generations and the older generations is an open question. A PEW survey for the US, clearly did show (Pew Research Center 2020) that key policy priorities – say the top 6 topics – differed much between old and young, except for the field of health care cost.

It is clear that economic globalization and certainly trade in intermediate products goes along with specific risk, partly related to logistics, partly related to an epidemic risk between the producing countries and the countries that are part of the logistics chain. With the Global Health Security Index the NTI/Johns Hopkins University research group has developed a very useful index which is not only important for understanding the quality of the health systems in most countries in the world but which could also be useful for a better understanding of the risk to international production chains and the risks faced by foreign investors in various host countries.

With airline passenger traffic coming to a nearly global stop in March 2020, the effective price for air freight is rising. This, of course, will concern only a small part of international trade, but some of the transported goods and intermediate products are crucial for the respective importing countries. Thus some additional negative supply-side spillover effects in the logistics sector could occur. Regions with big airports in most countries of the world are likely to face particular short-term economic problems in 2020. As regards innovation dynamics, one may expect that many firms under the simple heading of “organizing home office networking” will embark upon major innovation projects which, in the long term, could affect the world economy and might actually contribute to an economic upswing after the crisis. The natural winners of the global COVID-19 economic crisis should be companies in the sector of information & communication technology as well as telecommunications companies and firms in the news and digital entertainment sector; plus the health equipment sector and part of the pharmaceutical sector.

Every epidemic has four main economic challenges: a) how to minimize international and national diffusion; b) how to efficiently help those patients who are ill; c) how to quickly develop a vaccine that could help to make a very large part of society immune, for example against the relevant virus; d) how to fight the negative macroeconomic effects of the epidemic. As regards a) and b), countries which are in the medium range or the lower part of the index tables have every reason to work on improving their position in an international comparative perspective. Obviously, when it comes to benchmarking, the creation of different benchmarking groups could be useful, for example countries could be grouped according to the size of per capita income and the intensity of trade and foreign direct investment (relative to total investment).

The EU has a particular problem, namely that the ECB has little room to maneuver left. Hence, options for a coordinated fiscal policy and joint measures to rebuild international production networks quickly should be carefully studied. There is little doubt that lobbying against an effective anti-epidemic policy could be strong - influential soccer clubs in many EU countries, for example, can be expected to wage a fierce battle against playing lucrative soccer matches in empty stadiums; here the European Commission should develop clear principles that emphasize the authoritative role of experts and physicians with relevant specializations. As regards the role of the health systems in EU countries, it would be adequate to emphasize more strongly in the public political debate the many advantages of European type health insurance systems. This, of course, does not mean to overlook important opportunities to make health systems in EU countries more efficient and innovative. As regards cooperation with the US, more transatlantic city partnerships (twinning EU cities with cities in the US) could be useful; benchmarking the health care systems might be included as a field of comparison in such partnerships. With respect to a potential future free trade agreement between the EU and the US, it is not in the EU’s interest to allow US health care providers easy access to markets in the EU since this would certainly bring a strong tendency to raise hospital costs in Europe – this is not in the interest of people in the EU. In this perspective, the COVID-19 problem in the US will hopefully become a starting point for the US Administration as well as many state governments to reconsider carefully reform options in the health care sector.

As regards the EU(27), it should be useful to modernize health systems in many member countries with weak scores in the Global Health Security Index. This is not only in the interest of the respective countries but of all EU member countries and the world economy, respectively. Epidemic protection investment thus could become a new field of common co-financing in the EU. From an Economics perspective it is crucial to emphasize that protection against epidemics has partly elements of an international public good. This naturally makes adequate international cooperation – for example, in the EU, but indeed in the G20 and the world economy - necessary. It makes cooperation rather difficult if national economic policy, particularly health policy, is organized in a rather inconsistent way as is the case in Germany (Kaufmann 2009). From this perspective, the COVID-19 pandemic requires national policy reforms (in Germany including reforms at the level of the states which have a considerable responsibility in epidemic policy, while the ministry of health at the national level has fairly weak competences in fighting an epidemic). The fact that fighting the COVID-19 pandemic is a global public good raises special issues in terms of international cooperation and overcoming potential free rider problems, respectively.

As regards the European Union, one should expect that stronger efforts will be launched to develop vaccines against the coronavirus. It is not really clear why the morbidity in the COVID-19 epidemic in Italy seemed to be much higher in spring 2020 than in other EU countries; except that a high share of the elderly in the overall population and a rather low ratio of acute care beds to the population 65+ stood for two unfavorable indicators in the health system. Adding acute care beds in hospitals in the short and medium term will be a common challenge in many countries in OECD countries plus China as well as Newly Industrialized Countries and developing countries. It remains to be seen whether the specific British approach will work: The UK – according to government decisions in mid-March – does not want to follow the anti-epidemic policy of other OECD countries and instead favors a mildly controlled propagation of the coronavirus, while requiring the elderly to undergo a three months home quarantine.

Economic stabilization measures should put a focus on five key elements:

- Liquidity measures to shore up firms and banks facing serious coronavirus-related problems; since the tourism sector – in addition to manufacturing industry and industry-related services – consists typically of many small and medium-sized firms, the overall number of firm facing liquidity problems could be much higher than in previous major recessions.
- Fiscal policy measures which could mean more investment in health system modernization and back-up facilities to produce crucial chemical ingredients for pharmaceuticals. Postponing tax payments for firms and self-employed individuals could be crucial for maintain liquidity. The nationalization of big firms and banks should not be excluded, but firms and banks are responsible for also coming up with their own adjustment measures. It will be almost inevitable to pay transfers to certain groups of households which are strongly affected by the recession and the global economic slowdown.
- An expansionary monetary policy seems to be somewhat inadequate in the special setting of the pandemic – possibly except for China, namely assuming that the epidemic in China is over. There is, however, the risk that China could import the coronavirus from other countries or that a second coronavirus wave could emerge in China and the world economy.
- Multilateralism should be reinforced: There is clearly a role to be played by international organizations and broad cooperation in fighting the COVID-19 pandemic. However, as regards the EU and the EU countries’ reaction in mid-March 2020, with many national closing measures at the borders, one may argue that the pandemic has reinforced political and economic nationalism. There was little co-operation among EU member countries in the first quarter of 2020 – the role of the European Commission was rather modest. As regards the Eurozone, one should not rule out that the ESM, a special emergency fund created during the Euro crisis, would become active in the medium term once one or several Eurozone countries should face serious problems in access to finance to support government deficits and the rollover of government debt. Italy, as well as other EU countries with relatively high tourism output shares, might yet reach a position where this option could be useful. The Trump Administration, of course, stands for a problem here since its refusal of multilateralism and emphasis on bilateralism undermines both cooperation in health policy co-operation and in economic policy.
- The pandemic stands for a worldwide challenge and thus creates a rising demand for international information and access to relevant news. Public broadcasting should be opened up in the EU (and worldwide): Since national users have paid for the programming and the diffusion of TV and radio, one could easily offer on a reciprocal basis free international access to information/news programs. As regards Europe, this in turn could help to create a more unified EU public which could facilitate the consistent coordination of policymakers.

A new and broader benchmarking of health systems should be considered more by foreign investors worldwide: Locational competition in industry could focus more explicitly on health system aspects. The United States seems to face a particular problem of health system inefficiencies which could be remedied on the basis of more national and international benchmarking, particularly in the hospital sub-system. More comparative research is needed in both Europe, Asia and the US; and economists could certainly contribute in many ways, often teaming up with colleagues from the medical sciences, to stimulate both health system reform and more medium- and long-term stability. As the US and the EU – plus the UK – face growing competition from Asia and China, respectively, it could be useful for the EU and the US to cooperate in a better way which should include finding the optimal policy mix for economic stabilization as well as new ways for better health care.

The EU is facing a serious triple challenge in 2020: The coronavirus epidemic as a health care challenge, the ongoing internal conflicts regarding a potential new refugee wave – mainly linked to the Syrian civil war and the instability in Afghanistan – and a global output decline which is partly related to the pandemic. With the pending bankruptcy in Lebanon, there is an additional risk of a new refugee wave for the EU since about two million refugees live in the Lebanon. If economic and politic chaos should shape Lebanon in 2020, there could be massive refugee waves from the Lebanon to Turkey and from Turkey to the EU (plus some direct refugee moves from Lebanon to EU countries). Obviously, the European Commission is facing considerable challenges in 2020. The Commission should become more active in coordinating epidemic policies in the EU – it is strange that in some European countries soccer games are taking place behind closed doors without spectators because the risk of COVID-19 spreading is considered too high, but in early March 2020, several EU countries have allowed soccer games to go on as usual. The question of European soccer tournaments is also one important issue. Common standards in the epidemic policies of EU countries should be adopted and the EU should help developing countries as well as neighboring countries which face particular pandemic problems. Compared to the US, western EU countries – most countries in the Eurozone – seem to have relatively high quality health systems and, at the bottom line, the EU in the future should push for exporting the Social Market Economy and become more eager in adopting EU reforms.

While much emphasis is placed on the expenditure side in the US health policy debate over higher health care expenditures, the relevant economic policy perspective is different, namely how the ratio of health care expenditures relative to GDP is developing in the long run (GDP partly being determined by the production potential for which effective labor input is one crucial element to consider). This needs more attention in future research and policy: As the health insurance expenditure-GDP ratio positively affects effective labor input and hence GDP, a rise of the health insurance coverage (h) affects both the numerator and the denominator of that ratio. The health insurance-GDP ratio could fall if the output effect of a rise of h would be stronger than the effect in the numerator. Finally, if raising h – plus better efficiency-enhancing regulation for competition brings a higher life expectancy – there is still a positive welfare effect to be considered if individuals maximize lifetime consumption. The length of the lifetime period is endogenous and life expectancy levels in most western EU countries exceed that of the US by several years in the first two decades of the twenty-first century.

The coronavirus pandemic is likely to raise the demand for health insurance worldwide if conventional wisdom holds that insurance companies in a country with a hurricane shock will experience higher costs as insurance polices will have to be honored, at the same time peoples’ experience of the hurricane shock will stimulate the demand for insurance against hurricanes and raise the average willingness to pay – the hurricane event is like natural advertising for hurricane insurance (see, for the case of the September 11 attacks in the US, a related perspective in Wang and Corbett 2008). EU countries with very broad health insurance coverage might not see much change in their respective health insurance systems but rather police holders will enjoy a higher post-pandemic consumer rent as the implicit demand for health insurance becomes larger; private health insurance might benefit from this effect in the EU. In the US and many developing countries, as well as newly industrializing countries, the demand for health insurance could considerably rise in the medium term. As a final interesting structural shift effect, one may anticipate that the epidemic-imposed massive switch to more home office work could have a ratchet effect so that the post-pandemic demand for office space will decline (beyond the negative output-related effect) which will reduce the relative price for office buildings worldwide. In parallel, the demand for ICT technology and telecommunication services will strongly increase which, given the technology-intensity of ICT, could amount to a positive supply shock. Economic policymakers thus should be aware that restarting the economy after the peak of the pandemic does not mean simply to restart the “old economy”. For investors these reflections could point to rather easy opportunities for gains in the course of the adjustment dynamics.

As regards the global COVID-19 pandemic, one may argue that this amounts to an international symmetric economic shock which will generate some automatic parallel stabilization policy measures in many countries. However, it is was not really clear in March 2020 to what extent the pandemic is a strongly symmetric shock – the timing of the start of the epidemic differs across countries and the sectoral composition of output in certain countries is also different from that of other countries; and the country-specific response of the health system and health policy, respectively, also differs across countries. As regards cooperation among countries, this seems to be rather difficult and the Eurozone countries obviously had particular problems in finding agreement as to the degree to which strong cooperation and possibly the use of Eurobonds should be considered. Traditionally, there is resistance to Eurobonds in northern Eurozone countries, including Germany, while southern Eurozone countries – including Italy and Spain as countries rather strongly affected by the coronavirus shock in terms of a high case fatality rate – would rather push for this new option. As one can show, however, the coronavirus epidemic, coupled with a strong recession in the EU, the US and other countries, does not stand for the traditional Eurobond debate and the associated political setting: A key question concerns the extent to which such bonds could be launched with partial underlying collateral through gold and currency reserves in order to limit the liability risk of partner countries with rather low debt-GDP ratios (e.g. Germany or the Netherlands) and then about 40% of such Joint Eurobonds – JEBs – could be acquired by, say, the European Central Bank through its new pandemic quantitative easing program; such an approach, coupled with a special wealth taxation in Italy and Spain to make sure that the interest and principal of bonds can be paid without problems, should indeed be considered carefully as only this approach would firmly help to avoid a new Euro crisis and could mitigate an otherwise extremely negative economic shock in Italy and Spain (WELFENS 2020). Mitigating the economic shock in the first half of 2020 and making sure that a broad and sustained restart of the economies in Europe, Northern America and Asia and other world regions take place represent significant challenges for policymakers. As long as no vaccination is available, temporary lockdowns and “stay-at-home” periods will have to be used by certain countries such that a broad and strong international restart could prove difficult. It might also turn out to be the first major international crisis after 1945 without US leadership in fighting the crisis; a potential problem which I had pointed to previously – the lack of qualified staff in the Trump Administration is critical (WELFENS 2019). The US is the only OECD country where millions of people stand to lose not only their job in the recession but also their health insurance – in the midst of the coronavirus epidemic; one more reason to consider reforms in the US.

## Appendix 7 Regression Country List and Source Data

**Table:** 

Abb.	Variables	Measures	Units	Source
gdppc	GDP per capita	PPP (current international $)	$ millions	World Bank
ghs	GHS Index	overall score		NTI/Johns Hopkins (2019)
ifdi_trueo~n	Inward FDI true openness			World Bank
fdi_trueopen	FDI true openness			World Bank
	FDI inflows	US dollars at current prices	$ millions	World Bank
	FDI outflows	US dollars at current prices	$ millions	World Bank
trade_true~n	Trade true openness			World Bank
	Exports of goods and services	US dollars at current prices	$ millions	World Bank
	Imports of goods and service	US dollars at current prices	$ millions	World Bank
	GDP	US dollars at current prices	$ millions	World Bank
Country List				
Afghanistan	Dominican Republic	Libya	Seychelles	
Albania	Ecuador	Lithuania	Sierra Leone	
Algeria	Egypt	Luxembourg	Singapore	
Angola	El Salvador	Madagascar	Slovakia	
Antigua and Barbuda	Equatorial Guinea	Malawi	Slovenia	
Argentina	Estonia	Malaysia	Solomon Islands	
Armenia	Eswatini	Maldives	South Africa	
Australia	Ethiopia	Mali	South Korea	
Austria	Fiji	Malta	Spain	
Azerbaijan	Finland	Mauritania	Sri Lanka	
Bahamas	France	Mauritius	St. Kitts and Nevis	
Bahrain	Gabon	Mexico	St. Lucia	
Bangladesh	Gambia	Moldova	St. Vincent and The Grenadines	
Barbados	Georgia	Mongolia	Sudan	
Belarus	Germany	Montenegro	Suriname	
Belgium	Ghana	Morocco	Sweden	
Belize	Greece	Mozambique	Switzerland	
Benin	Grenada	Myanmar	Tajikistan	
Bhutan	Guatemala	Namibia	Tanzania	
Bolivia	Guinea	Nepal	Thailand	
Bosnia and Herzegovina	Guinea-Bissau	Netherlands	Timor-Leste	
Botswana	Guyana	New Zealand	Togo	
Brazil	Haiti	Nicaragua	Tonga	
Brunei Darussalam	Honduras	Niger	Trinidad and Tobago	
Bulgaria	Hungary	Nigeria	Tunisia	
Burkina Faso	Iceland	North Macedonia	Turkey	
Burundi	India	Norway	Tuvalu	
Cabo Verde	Indonesia	Oman	Uganda	
Cambodia	Iraq	Pakistan	Ukraine	
Cameroon	Ireland	Panama	United Arab Emirates	
Canada	Israel	Papua New Guinea	United Kingdom	
Central African Republic	Italy	Paraguay	United States	
Chad	Jamaica	Peru	Uruguay	
Chile	Japan	Philippines	Uzbekistan	
China	Jordan	Poland	Vanuatu	
Colombia	Kazakhstan	Portugal	Vietnam	
Comoros	Kenya	Qatar	Yemen	
Congo, Rep.	Kiribati	Romania	Zambia	
Congo, Dem. Rep.	Kuwait	Russia	Zimbabwe	
Costa Rica	Kyrgyz Republic	Rwanda		
Cote d’Ivoire	Laos	Samoa		
Croatia	Latvia	Sao Tome and Principe		
Czech Republic	Lebanon	Saudi Arabia		
Denmark	Lesotho	Senegal		
Dominica	Liberia	Serbia		

## Figures and Tables

**Table 10:**
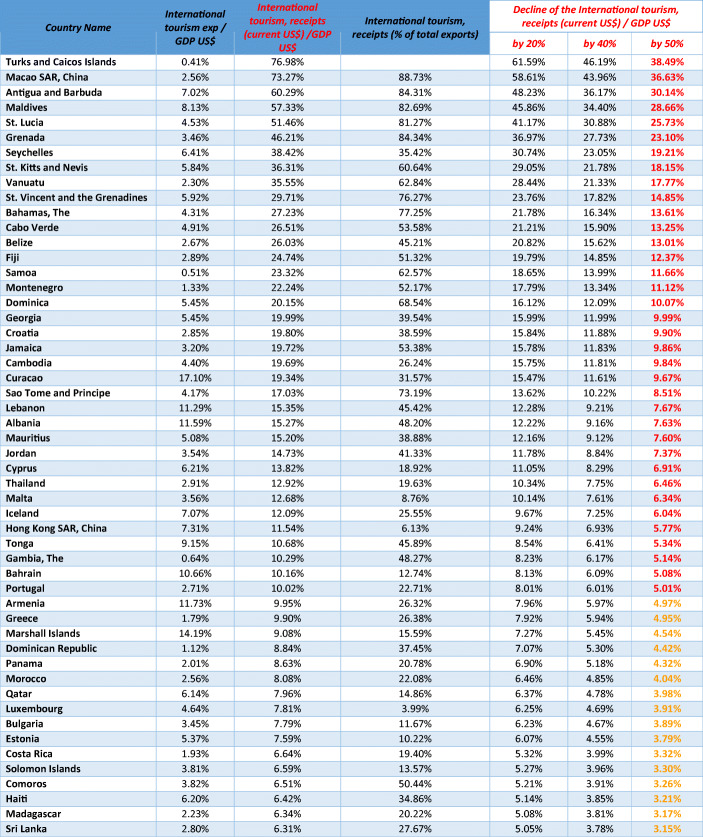

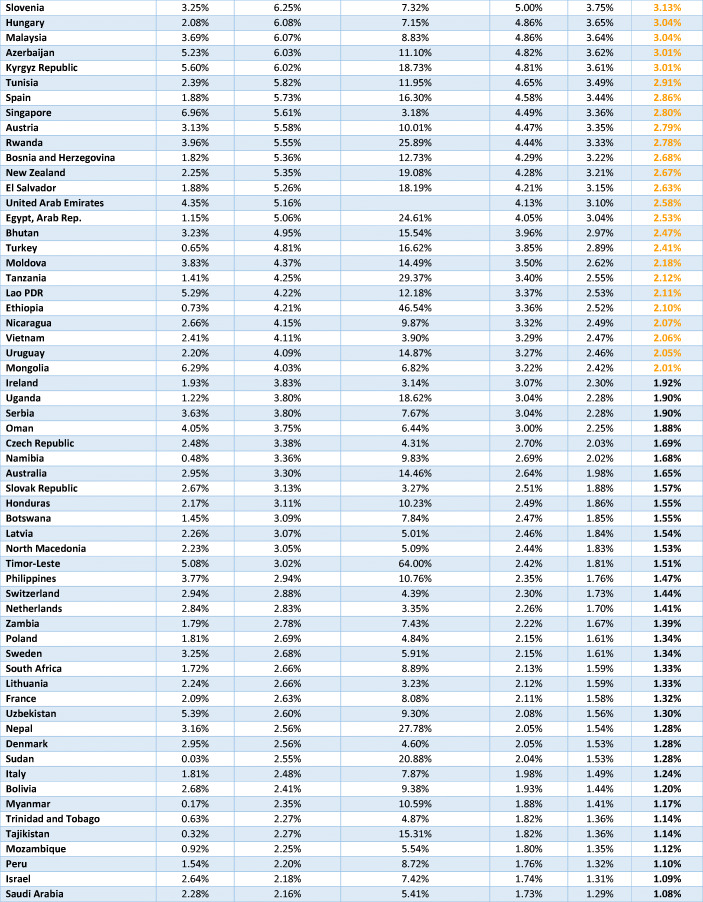

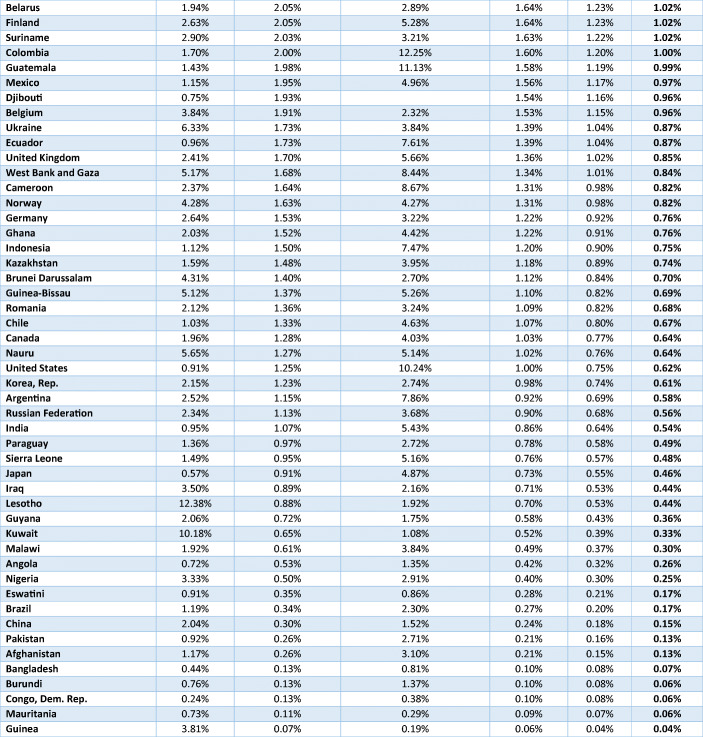
International Tourism Receipts as Percentage of GDP and Output Decline

Source: Own representation of data from the World Development Indicators

**Table 11:**
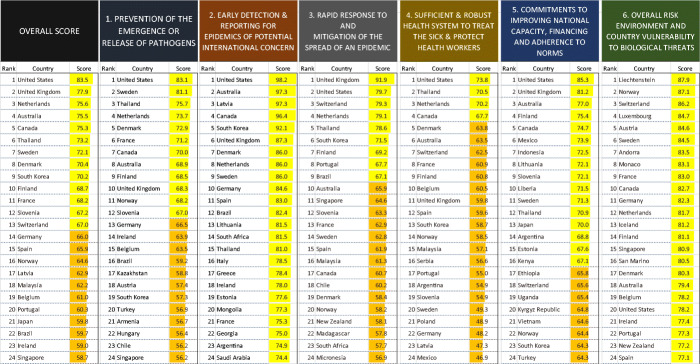

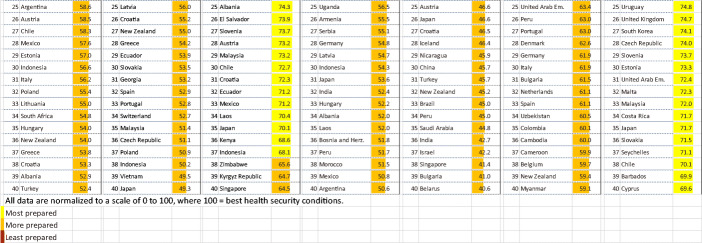
Global Health Security Index, Results Top 40 by Overall Score and Individual Indicators

Source: Global Health Security Index, NTI/Johns Hopkins University (2019)

**Fig. 13:**
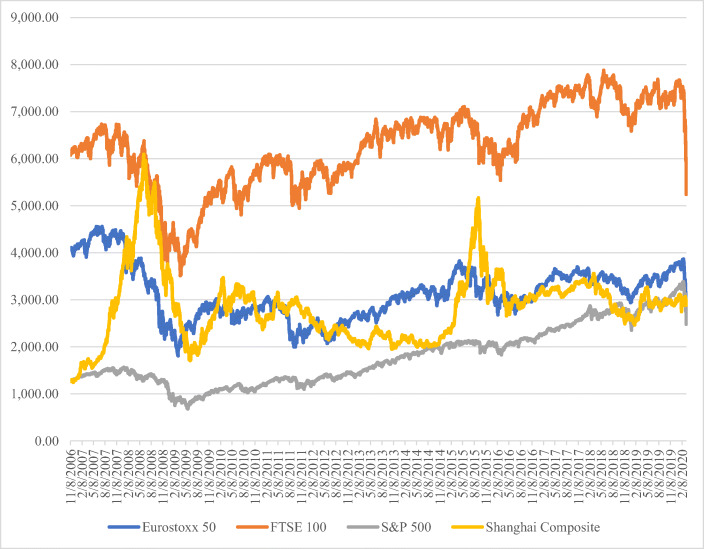
Developments on Major Global Stock Market Indices, 08.11.2006–16.03.2020. Source: Own representation of data available from finanzen.net

## Appendix 1 World Health Organization COVID-19 Statistics

**Table 7:** World Health Organization Statistics on COVID-19 in Chinese Regions as of 9 March 2020

	Confirmed cases	Deaths
Hubei	67743	3007
Guangdong	1352	8
Henan	1272	22
Zhejiang	1215	1
Hunan	1018	4
Anhui	990	6
Jiangxi	935	1
Shandong	758	6
Jiangsu	631	0
Chongqing	576	6
Sichuan	539	3
Heilongjiang	481	13
Beijing	428	8
Shanghai	342	3
Hebei	318	6
Fujian	296	1
Guangxi	252	2
Shaanxi	245	1
Yunnan	174	2
Hainan	168	6
Guizhou	146	2
Tianjian	136	3
Shanxi	133	1
Liaoning	125	1
Gansu	124	2
Hong Kong SAR	114	3
Jilin	93	1
Xinjiang	76	3
Inner Mongolia	75	1
Ningxia	75	0
Taipei	45	1
Qinghai	18	0
Macao SAR	10	0
Xizang	1	0
Total	**80904**	**3124**

Source: Own representation of data available from the WHO, Coronavirus Situation Dashboard, figures as of 9 March 2020 https://www.who.int/emergencies/diseases/novel-coronavirus-2019

**Table 8:** World Health Organization Statistics on COVID-19, Number of Infected Persons for Selected Countries as of 9 March 2020

Countries	Number of Persons Infected
China	80904
Republic of Korea	7382
Italy	7375
Iran (Islamic Republic of)	6566
France	1116
Germany	902
Spain	589
Japan	488
Switzerland	332
United Kingdom	277
Netherlands	256
United States	213
Sweden	203
Belgium	200
Austria	102
Australia	77
Greece	73
Canada	60
India	39
Denmark	36
Brazil	25
Ireland	21
Saudi Arabia	15
Argentina	12
Mexico	7
Russian Federation	7
Indonesia	6
South Africa	3
Nigeria	1

Source: Own representation of data available from the WHO, Coronavirus Situation Dashboard, figures as of 9 March 2020 https://www.who.int/emergencies/diseases/novel-coronavirus-2019 It should be noted that Turkey had yet to notify the WHO or officially confirm any cases of COVID-19 infection or deaths in the country

## Appendix 2 Tourism in a Chinese Perspective

China has had an increasing number of foreign arrivals and the number of Chinese outbound travelers has also increased – indeed, it has almost quadrupled between 2009 and 2018. As regards the earnings from domestic tourism relative to GDP, the share has increased from 2.9% in 2009 to 5.7% and 5.5% in 2017 and 2018, respectively. From this perspective, travelling restrictions at the national or international level for Chinese tourists could have considerable negative effects, both within China and in the primary destination countries for Chinese tourists. While earnings from domestic tourism is not the same as value-added, it is clear that the strong decline of domestic tourism would already effect China’s aggregate output considerably. A decline of, for example, 50% in 2020 could reduce Chinese GDP by about two percentage points and this in turn could have negative spillover effects to the Eurozone/EU and the US.

## Appendix 3

**Table 9:** Travel Receipts and Expenditures in Balance of Payments, Selected Countries, 2013 and 2018

	Receipts		Relative to GDP	Expenditures (million EUR)		Relative to GDP	Balance (million EUR)
	(million EUR)		2018 (%)			2018 (%)	
	2013	2018		2013	2018		2018
EU-27 (¹)	126010.7	158148.2	1.17	86737.8	109245.4	0.81	48902.7
Belgium	10074	7548	1.64	16692	15687	3.41	-8139
Bulgaria	2890.8	3822.5	6.82	840	1584.4	2.82	2238.1
Czechia	5303.1	6312.6	3.04	3493.8	5055.5	2.44	1257.1
Denmark	5385.1	7710.4	2.56	7584.1	8887.5	2.95	-1177.1
Germany	31081	36390	1.09	68793	80933	2.42	-44543
Estonia	1255.8	1515.7	5.82	802.8	1245.5	4.78	270.2
Ireland	3370	5237	1.62	4669	6270	1.93	-1033
Greece	12152	16086	8.71	1835	2191	1.19	13895
Spain	51589	69023	5.74	12359	22692	1.89	46331
France	53103	55450	2.36	31787	40528	1.72	14922
Croatia	6135.9	9488.6	18.38	679.3	1434.2	2.78	8054.4
Italy	33063	41712	2.36	20309	25484	1.44	16228
Cyprus	2211	2940	13.91	944	1315	6.22	1625
Latvia	651	897	3.08	538	660	2.26	237
Lithuania	1035.2	1274.2	2.82	805	1185.5	2.62	88.7
Luxembourg	3797	4230	7.04	2422	2731	4.55	1499
Hungary	4042.8	5850.4	4.37	1437.1	2238.6	1.67	3611.7
Malta	1057.2	1573.8	12.71	288.8	440.5	3.56	1133.3
Netherlands	10343	15236	1.97	15589	17956	2.32	-2720
Austria	15237	19559	5.07	7738	10143	2.63	9416
Poland	8549.1	11911.5	2.40	6646.4	8249.6	1.66	3661.9
Portugal	:	16840	8.26	:	4662	2.29	12178
Romania	1391.6	2876.2	1.41	1507.7	4522.2	2.21	-1646
Slovenia	2093.5	2704.1	5.91	1068.3	1389.5	3.04	1314.6
Slovakia	1997.7	2709.8	3.02	1782	2225.4	2.48	484.5
Finland	3044	3102	1.32	3989	5151	2.20	-2049
Sweden	8181.8	12651.3	2.68	11551.6	15293.4	3.25	-2642.1
United Kingdom	34675.6	41167.5	1.70	45854.5	58442.9	2.41	-17275.4
Iceland	813	2657.8	12.09	638.9	1553.8	7.07	1104
Norway	:	4956.2	:	:	14699.4	:	-9743.2
Switzerland	12644	14370.4	3.91	12163.3	15539.5	4.22	-1169.1
Montenegro	666	1001	0.17	37	58	0.01	943
North Macedonia	200.8	324.8	6.97	98.3	219.8	4.71	105
Albania	:	1855.7	17.35	:	1426.1	13.33	429.6
Serbia	792	1317	10.30	841	1396	10.92	-79
Turkey	21089.4	21482.1	50.13	3623.3	3888.2	9.07	17593.9
Bosnia and Herzegovina	516.5	875.8	0.13	100.6	221.3	0.03	654.4
Kosovo*	647.5	1228.2	7.33	135.3	302.3	1.80	925.9

Source: Eurostat, Industry and Services, Tourism - Table 3: Travel receipts and expenditure in balance of payments, 2013–2018

## Appendix 6 Health System Comparison, US and Western Europe

The figure above, based on WELFENS (2019), shows the variation between health care expenditures (expressed as a percentage of gross domestic product) and estimated life expectancies. One can clearly see a significant difference between the US and western European countries (selected EU member states and Switzerland). The cause for this is the higher spending on health care and lower life expectancy in the use, raising questions about the efficiency of the US health care system.

International coordination of the US in the field health policy has been weakened by internal decisions of the Trump Administration. One feature of the US Trump Administration is that the senior director, Rear Admiral Timothy Ziemer, of Global Health and Biodefense on the National Security Council left the Trump Administration in May 2018. In a letter to the National Security Adviser Robert O’Brien, dated February 18, 2020, a group of 27 senators proposed that a new global health security expert should be appointed to the NSC. On February 26, 2020, President Trump appointed Vice President Pence to head the anti-pandemic efforts of the Administration relating to COVID-19.

## Appendix 9 Condition Under Which Health Insurance Density is Neutral with Respect to the Level of the Growth Path

Consider the equation:

1’ $$ y\#={h}^{\ss \prime }{\left[\frac{\left(s\left(1-t\prime \prime \right)\left(1-\alpha \ast \ss \right)+s\prime \alpha \ast \ss \right)}{\left(a+n\prime \left(1\hbox{--} C\prime \prime \left(1-h\prime \prime h\right)\right)+d\prime \right)}\right]}^{\frac{\ss }{\left(1-\ss \right)}} $$

2’ $$ {\displaystyle \begin{array}{l}\frac{dy\#}{dh}=\beta \hbox{'}{h}^{\ss \prime -1}{\left[\frac{\left(s\left(1-t\prime \prime \right)\left(1-\alpha \ast \ss \right)+s\prime \alpha \ast \ss \right)}{\left(a+n\prime \left(1\hbox{--} C\prime \prime \left(1-h\prime \prime h\right)\right)+d\prime \right)}\right]}^{\frac{\ss }{\left(1-\ss \right)}}\\ {}\operatorname{}-\frac{\beta }{1-\beta }{h}^{\ss \prime }{\left[\frac{\left(s\left(1-t\prime \prime \right)\left(1-\alpha \ast \ss \right)+s\prime \alpha \ast \ss \right)}{\left(a+n\prime \left(1\hbox{--} C\prime \prime \left(1-h\prime \prime h\right)\right)+d\prime \right)}\right]}^{\frac{\ss }{\left(1-\ss \right)}-1}\frac{\left(s\left(1-t^{\prime\prime}\right)\left(1-\alpha \ast \ss \right)+s^{\prime}\alpha \ast \ss \right)n^{\prime }C^{\prime\prime }h^{\prime\prime }}{{\left(a+n'\left(1\hbox{--} C"\left(1-h"h\right)\right)+d'\right)}^2}\end{array}} $$

3’ $$ {\displaystyle \begin{array}{l}\frac{dy\#}{dh}=0\kern0.36em \Rightarrow h=0\vee \beta \hbox{'}{\left[\frac{\left(s\left(1-t\prime \prime \right)\left(1-\alpha \ast \ss \right)+s\prime \alpha \ast \ss \right)}{\left(a+n'\left(1\hbox{--} C"\left(1-h"h\right)\right)+d'\right)}\right]}^{\frac{\ss }{\left(1-\ss \right)}}\\ {}\operatorname{}-\frac{\beta }{1-\beta }{\left[\frac{\left(s\left(1-t\prime \prime \right)\left(1-\alpha \ast \ss \right)+s\prime \alpha \ast \ss \right)}{\left(a+n\prime \left(1\hbox{--} C\prime \prime \left(1-h\prime \prime h\right)\right)+d\prime \right)}\right]}^{\frac{\ss }{\left(1-\ss \right)}-1}\frac{\left(s\left(1-t^{\prime\prime}\right)\left(1-\alpha \ast \ss \right)+s^{\prime}\alpha \ast \ss \right)n\hbox{'}C\hbox{'}\hbox{'}h\hbox{'}\hbox{'}}{{\left(a+n\prime \left(1\hbox{--} C\prime \prime \left(1-h\prime \prime h\right)\right)+d\prime \right)}^2}=0\end{array}} $$

Assuming β = 1/3rd (as a typical assumption for OECD countries):

4’ $$ {\displaystyle \begin{array}{l}\operatorname{}\Rightarrow \beta \hbox{'}{\left[\frac{\left(s\left(1-t\prime \prime \right)\left(1-\alpha \ast \ss \right)+s\prime \alpha \ast \ss \right)}{\left(a+n\prime \left(1\hbox{--} C\prime \prime \left(1-h\prime \prime h\right)\right)+d\prime \right)}\right]}^{\frac{1}{2}}=\frac{\frac{1}{2}\frac{\left(s\left(1-t^{\prime\prime}\right)\left(1-\alpha \ast \ss \right)+s^{\prime}\alpha \ast \ss \right)n^{\prime }C\hbox{'}\hbox{'}h\hbox{'}\hbox{'}}{{\left(a+n\prime \left(1\hbox{--} C\prime \prime \left(1-h\prime \prime h\right)\right)+d\prime \right)}^2}}{{\left[\frac{\left(s\left(1-t\prime \prime \right)\left(1-\alpha \ast \ss \right)+s\prime \alpha \ast \ss \right)}{\left(a+n\prime \left(1\hbox{--} C\prime \prime \left(1-h\prime \prime h\right)\right)+d\prime \right)}\right]}^{\frac{1}{2}}}\\ {}\operatorname{}\iff \beta \hbox{'}\frac{\left(s\left(1-t^{\prime\prime}\right)\left(1-\alpha \ast \ss \right)+s^{\prime}\alpha \ast \ss \right)}{\left(a+n^{\prime}\left(1\hbox{--} C^{\prime\prime}\left(1-h^{\prime\prime }h\right)\right)+d^{\prime}\right)}=\frac{1}{2}\frac{\left(s\left(1-t^{\prime\prime}\right)\left(1-\alpha \ast \ss \right)+s^{\prime}\alpha \ast \ss \right)n\hbox{'}C\hbox{'}\hbox{'}h\hbox{'}\hbox{'}}{{\left(a+n\prime \left(1\hbox{--} C\prime \prime \left(1-h\prime \prime h\right)\right)+d\prime \right)}^2}\\ {}\operatorname{}\iff \beta \hbox{'}\left(s\left(1-t^{\prime\prime}\right)\left(1-\alpha \ast \ss \right)+s^{\prime}\alpha \ast \ss \right)=\frac{1}{2}\frac{\left(s\left(1-t^{\prime\prime}\right)\left(1-\alpha \ast \ss \right)+s^{\prime}\alpha \ast \ss \right)n\hbox{'}C\hbox{'}\hbox{'}h\hbox{'}\hbox{'}}{\left(a+n^{\prime}\left(1\hbox{--} C^{\prime\prime}\left(1-h^{\prime\prime }h\right)\right)+d^{\prime}\right)}\\ {}\operatorname{}\iff \beta \hbox{'}=\frac{n\hbox{'}C\hbox{'}\hbox{'}h\hbox{'}\hbox{'}}{2\left(a+n^{\prime}\left(1\hbox{--} C^{\prime\prime}\left(1-h^{\prime\prime }h\right)\right)+d^{\prime}\right)}\\ {}\operatorname{}\iff 2\beta \hbox{'}a+2\beta \hbox{'}n^{\prime}\left(1\hbox{--} C^{\prime\prime}\left(1-h^{\prime\prime }h\right)\right)+2\beta \hbox{'}d^{\prime }=n\hbox{'}C\hbox{'}\hbox{'}h\hbox{'}\hbox{'}\\ {}\operatorname{}\iff 2\beta^{\prime }n'C\hbox{'}\hbox{'}h\hbox{'}\hbox{'}h=n\hbox{'}C\hbox{'}\hbox{'}h\hbox{'}\hbox{'}-2\beta \hbox{'}\left(a+n^{\prime }-n^{\prime }C\hbox{'}\hbox{'}+d^{\prime}\right)\\ {}\operatorname{}\iff -2\beta \hbox{'}n^{\prime }C\hbox{'}\hbox{'}h\hbox{'}\hbox{'}h+2\beta \hbox{'}n^{\prime }C\hbox{'}\hbox{'}+n\hbox{'}C\hbox{'}\hbox{'}h\hbox{'}\hbox{'}=2\beta \hbox{'}\left(a+n^{\prime }+d^{\prime}\right)\\ {}\operatorname{}\iff C\hbox{'}\hbox{'}\left[n\hbox{'}\left(2\beta \hbox{'}+h\hbox{'}\hbox{'}-2\beta \hbox{'}h\hbox{'}\hbox{'}h\right)\right]=2\beta \hbox{'}\left(a+n^{\prime }+d^{\prime}\right)\\ {}\kern2.5em \iff C\hbox{'}\hbox{'}=\frac{2\beta \hbox{'}\left(a+n^{\prime }+d^{\prime}\right)}{n\hbox{'}\left[2\beta \hbox{'}\left(1-h\hbox{'}\hbox{'}h\right)+h\hbox{'}\hbox{'}\right]}\end{array}} $$

5’ $$ {\displaystyle \begin{array}{l}\iff h=\frac{n\hbox{'}C\hbox{'}\hbox{'}h\hbox{'}\hbox{'}-2\beta \hbox{'}\left(a+n'-n'C\hbox{'}\hbox{'}+d'\right)}{2\beta \hbox{'}n'C\hbox{'}\hbox{'}h\hbox{'}\hbox{'}}\\ {}\iff h=\frac{1}{2\beta \hbox{'}}-\frac{a+n'-n'C\hbox{'}\hbox{'}+d'}{n'C\hbox{'}\hbox{'}h\hbox{'}\hbox{'}}\\ {}\iff h=\frac{1}{2\beta \hbox{'}}-\frac{a+d'}{n'C\hbox{'}\hbox{'}h\hbox{'}\hbox{'}}+\frac{1}{h\hbox{'}\hbox{'}}-\frac{1}{C\hbox{'}\hbox{'}h\hbox{'}\hbox{'}}\end{array}} $$

The implication is that for $\frac{dy\#}{dh}=0$ a specific size C“should be relevant or that – given C“– a specific set of parameters must hold.

## Appendix 10 Health Insurance Coverage (Percentage of Population) for Selected Countries, Public and Private

**Table 12:** Health Care Coverage in Selected Countries, Government/Social Health Insurance and Private Health Insurance

Health care coverage														
	Government/social health insurance							Private health insurance						
	Total health care							Total private health insurance (PHI) coverage						
Year	2012	2013	2014	2015	2016	2017	2018	2012	2013	2014	2015	2016	2017	2018
Australia	100.00	100.00	100.00	100.00	100.00	100.00	100.00	54.20	54.80	55.40	55.80	55.50	54.90	54.20
Canada	100.00	100.00	100.00	100.00	100.00	100.00	100.00	67.00	67.00	67.00	67.00	67.00	67.00	67.00
Denmark	100.00	100.00	100.00	100.00	100.00	100.00	100.00	27.10	28.80	31.70	32.00	32.50	29.20	
Finland	100.00	100.00	100.00	100.00	100.00	100.00	100.00	17.10	17.80	18.30	20.20	21.00	21.60	22.00
Ireland	100.00	100.00	100.00	100.00	100.00	100.00	100.00	45.70	44.40	43.60	45.30	45.40	45.40	
Israel	100.00	100.00	100.00	100.00	100.00	100.00	100.00	80.30	82.90	83.90	83.40	83.10	85.00	
Latvia						100.00	100.00	8.10	12.70	13.90	14.20	14.60	16.00	18.20
New Zealand	100.00	100.00	100.00	100.00	100.00	100.00	100.00	30.50	29.80	29.30	28.80	28.70	28.60	28.40
Norway	100.00	100.00	100.00	100.00	100.00	100.00	100.00							
Czechia	100.00	100.00	100.00	100.00	100.00	100.00		0.00	0.00	0.20	0.00	0.00	0.00	
Greece				86.00	100.00	100.00		12.50	12.50	11.50	12.00			
Italy	100.00	100.00	100.00	100.00	100.00	100.00								
Korea	100.00	100.00	100.00	100.00	100.00	100.00		56.50	61.00	63.20	66.80	67.70	67.60	
Portugal	100.00	100.00	100.00	100.00	100.00	100.00		20.20	21.00	22.30	25.70	26.10	26.60	
Slovenia	100.00	100.00	100.00	100.00	100.00	100.00		72.80	73.20	77.00	87.10	84.30	85.70	88.70
Sweden	100.00	100.00	100.00	100.00	100.00	100.00								
Switzerland	100.00	100.00	100.00	100.00	100.00	100.00		27.90					28.50	
United Kingdom	100.00	100.00	100.00	100.00	100.00	100.00		10.80	10.60	10.40	10.50	10.40	10.40	
Japan	100.00	100.00	100.00	100.00	100.00	100.00								
France	99.90	99.90	99.90	99.90	99.90	99.90	99.90	95.00		95.50				
Austria	99.90	99.90	99.90	99.90	99.90	99.90		34.50	35.20	35.70	36.20	36.50	36.90	
Netherlands	99.70	99.80	99.80	99.80	99.90	99.90		88.00	85.70	84.50	84.10	84.30	84.10	
Russia	99.80	99.20	99.90	100.00	100.00	99.70	99.60	11.60	11.50	11.30	10.40			
Iceland	99.80	99.80	99.80	99.70	99.60	99.50	99.60	0.20	0.20	0.20	0.30	0.40	0.50	0.40
Turkey	98.20	98.00	98.40	98.40	98.20	99.20		5.50	5.60	5.80	5.40	7.60	6.70	
Spain			99.10			99.00	100.00			15.60			16.50	
Belgium	99.00	99.00	99.00	99.00	99.00	98.70		79.70	80.40	82.20	83.60	84.90	84.40	
Colombia	96.40	96.00	96.60	97.60	95.70	94.90	94.70			4.20	7.80	6.90	8.20	
Slovak Rep.	95.00	94.60	94.20	93.80	94.50	94.60		0.00	0.00	0.00	0.00	0.00	0.00	
Estonia	93.70	93.60	93.90	94.00	94.00	94.10	94.50							
Hungary	96.00	96.00	95.00	95.00	95.00	94.00		0.00	0.00	0.00	0.00	0.00	0.00	
Lithuania	91.90	91.80	92.00	92.40	92.50	93.90	98.10				1.50	1.50	1.30	1.80
Poland	91.00	91.60	91.30	91.00	91.50	92.60		0.00	0.00	0.00	0.00	0.00	0.00	
Germany	88.80	89.00	89.10	89.20	89.30	89.40		33.00	33.00	33.80	33.90	33.90	34.30	
Mexico	89.60	91.60	92.90	92.30	89.80	89.30		7.60	7.50	7.70	7.60	8.10	8.70	
Chile	76.30	75.90	75.20	73.20	74.40	75.60	76.10							
United States	32.60	33.00	34.50	35.60	36.30	35.90		60.30	60.10	61.60	62.90	63.00	62.90	

Source: Own representation of data available from OECDStat, Social Protection

## Footnotes

1 It should be noted that by March 8, Turkey, for example, had yet had to notify the World Health Organization of any COVID-19 infections; the absence of the virus in the country is improbable (indeed, see figures eventually released in mid-March)
